# San Bernardino Cave (Italy) and the Appearance of Levallois Technology in Europe: Results of a Radiometric and Technological Reassessment

**DOI:** 10.1371/journal.pone.0076182

**Published:** 2013-10-16

**Authors:** Andrea Picin, Marco Peresani, Christophe Falguères, Giulia Gruppioni, Jean-Jacques Bahain

**Affiliations:** 1 Neanderthal Museum, Mettmann, Germany; 2 Institut Català de Paleoecologia Humana i Evolució Social (IPHES), Tarragona, Spain; 3 Universitat Rovira I Virgili, Àrea de Prehistòria, Tarragona, Spain; 4 Universitá di Ferrara, Dipartimento di Studi Umanistici, Sezione di Scienze Preistoriche e Antropologiche, Ferrara, Italy; 5 Département de Préhistoire du Muséum National d’Histoire Naturelle, UMR7194, Paris, France; University of Florence, Italy

## Abstract

The introduction of Levallois technology in Europe marked the transition from the Lower to the early Middle Paleolithic. This new method of flake production was accompanied by significant behavioral changes in hominin populations. The emergence of this technological advance is considered homogeneous in the European archaeological record at the Marine isotopic stage (MIS) 9/MIS 8 boundary. In this paper we report a series of combined electron spin resonance/U-series dates on mammal bones and teeth recovered from the lower units of San Bernardino Cave (Italy) and the technological analyses of the lithic assemblages. The San Bernardino Cave has yielded the earliest evidence of Levallois production on the Italian Peninsula recovered to date. In addition to our results and the review of the archaeological record, we describe the chronological and geographical differences between European territories and diversities in terms of technological developments. The belated emergence of Levallois technology in Italy compared to western Europe corresponds to the late Italian Neanderthal speciation event. The new radiometric dates and the technological analyses of San Bernardino Cave raise the issue of the different roles of glacial refugia in the peopling and the spread of innovative flaking strategies in Europe during the late Middle Pleistocene.

## Introduction

The introduction of the Levallois method in Europe is considered the technological innovation that marked the beginning of the Middle Paleolithic. As opposed to earlier flaking methods established by out-of-Africa migrations [1], this new concept of core configuration originated in the reorganization of local technologies. The emergence was gradual in the archaeological record and coexisted for a long time with previous technical traditions. This concurrence has been firstly interpreted on the base of typological association with retouched artifacts [2], [3]. Besides Acheulean industries with handaxes, the lithic assemblages without handaxes have been categorized as Clactonian [4]–[7] or Tayacian [8]–[12] for the association respectively with large notching-tools or Tayac points (convergent denticulates). This typological discrimination has been abandoned after the technological analysis of some lithic series that highlighted the similarities in the flaking strategies between the Acheulean and the Clactonian [13], [14]. Conversely the abundant component of denticulates and notching-tools in Tayacian has been questioned and associated with taphonomic processes rather than with real production patterns [8], [15], [16]. The production of Tayacian and Quinson points was inferred as part of the Acheulean toolkit and no more as fossile directeur. In this panorama the introduction of Levallois was interpreted as an internal development of the Acheulean and named as Acheulean *tardif* or *évoluées* [17]. However this definition understated the variability of the lithic collections in this chronological interval, in which Acheulean industries with/without handaxes coexisted with Pre-Mousterian industries characterized by a proper use of the Levallois technology [18], [19].

The overlapping of different techno-complexes has made it very difficult to explain the technological shifts in terms of the current paradigm of evolutionary advances in hominin cognitive capacities [20]. In fact, the appearance of Levallois technology was not a simple modification in flake production, but was part of more wide-sweeping behavioral changes that included the habitual use of fire [21], the manipulation of pigments [22], the mastery of hafting [23] and more elaborate hunting strategies [24]. Determining the timing of the appearance of this technological improvement is crucial to tracing when these new social adaptations took place and how they correlate with other behaviors considered symbolic in later periods [25], [26].

Some authors have argued that the Levallois method emerged uniformly in Europe in the late Middle Pleistocene [27]–[29]. Although the chronologies of the introduction of the Levallois method are well established in northern Europe and southern France, there is a certain degree of disagreement with regard to the Mediterranean area. Uncertainty about the ages of some sites and the interpretation of several lithic series has contributed to the exclusion of the Italian Peninsula from the debate on the European emergence of the Mousterian tradition. Our focus in this paper is to review the archaeological evidence in this temporal interval and present new radiometric and technological data from units VIII and VII of San Bernardino Cave (Italy). The results provide additional information relevant to understanding regional diversity in the production, establishment and spread of the technological innovation that marked an important advance in the technical behavior of Neanderthals.

### Chronology of the Emergence of the Levallois in Europe

**Figure 1:**
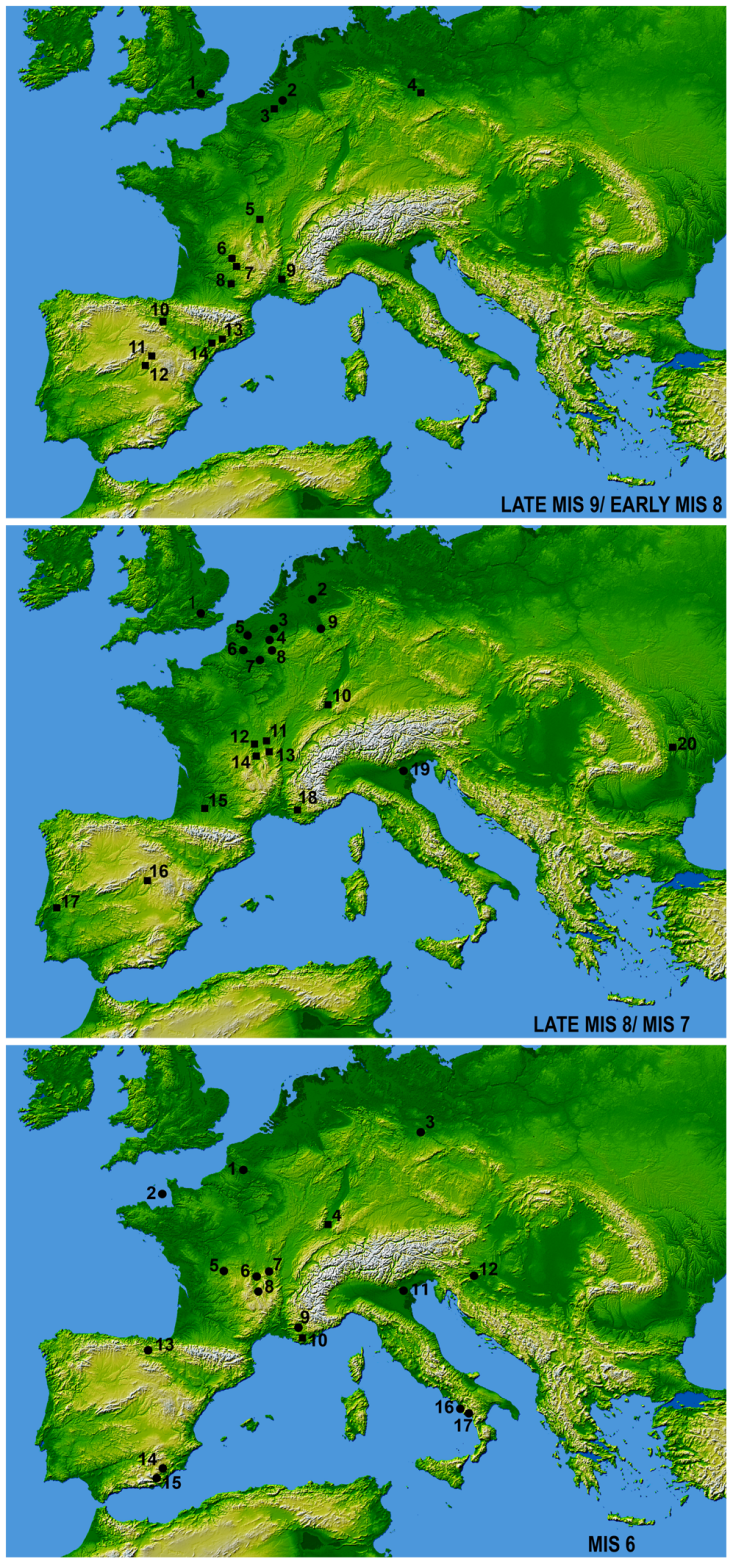
Maps of the archaeological sites discussed in the text. **A**: 1. Purfleet, 2. Mesvin IV, 3. Kesselt-Op De Schans, 4. Markkleeberg, 5. La Micoque, 6. Petit Bost, 7. Le Bosses, 8. Raspide 2, 9. Orgnac 3, 10. AT Gran Dolina, 11. Ambrona, 12. Aridos 1,13. Domeny, 14. Puig d’en Roca III; **B**: 1. Thames Valley, 2. Maastricht-Belvedere, 3. Biache-Saint-Vaast, 4. Bapaume Les Osiers, 5. Salouel, 6. Le Pucheuil, 7. Therdonne, 8. Le Rissori, 9. Ariendorf 1, 10. Achenheim, 11. Petit Bost, 12. Gran Rois, 13. Cantalouette, 14. Barbas, 15. Campsas, 16. Torralba, 17. Galeria Pesada, 18. Baume Bonne, 19. San Bernardino, 20. Korolevo; **C**: 1. Le Pucheuil, 2. La Cotte San Brelade, 3. Markkleeberg, 4. Achenheim, 5. Abri Suard, 6. Barbas, 7. Cantalouette, 8. Grotte Vaufrey, 9. Baume Bonne, 10. Lazaret, 11. San Bernardino, 12. Krapina, 13. Lezetxiki, 14. Cariguela, 15. Solana del Zamborino, 16. Poggio, 17. Molare. (Square: handaxe and Levallois; Circle: Levallois) (Base map from NASA http://www2.jpl.nasa.gov/srtm/world.htm).

The beginning of the use of the Levallois method has been dated to the end of MIS 9 and the beginning of MIS 8 (Figure 1A) in northern Europe at Purfleet (UK), Mesvin IV and Kesselt-Op de Schans (Belgium), Markkleeberg (Germany), in southern France at Orgnac 3, Les Bosses, Raspide 2, Petit Bost level 2 and upper levels of La Micoque, and on the Iberian Peninsula in unit TD10.1 of Gran Dolina, the upper member of Ambrona, Aridos 1, Domeny and Puig den Roca III. Handaxe production is present at all of these sites, except at Purfleet and Kesselt-Op de Schans (Bibliographic references are listed in Dataset S1).

The Iberian sites of Gran Dolina unit TD10.1, Ambrona Upper Member and Aridos 1 are normally associated with this temporal interval, although other dates have been proposed. At Gran Dolina, ESR/U-series [30] and TL/IRSL [31] dating place the appearance of Mode 3 between 300 and 400 ka and between 240 and 480 ka, respectively. The Upper Member complex of Ambrona has been dated by means of U/Th series at < 350 ka [32] and by ESR/U-series at 336 ± 72 ka [33]. Aridos 1, meanwhile, has been dated based on its micromammal assemblage at between 350 and 300 ka [34].

In northern Europe, the Levallois method reoccurred at the bridge between MIS 8 and MIS 7 (Figure 1B) in the Thames valley at Yiewsley Area and Creffield Road, Baker’s Hole, Ebbsfleet and the Lion Pit Tramway Cutting, and at Salouel and units A-C of Le Pucheuil in northern France. During early MIS 7, the Levallois method was present at Maastricht-Belvédère, Le Rissori, level IIa of Biache-Saint-Vaast, and at the end of MIS 7 at unit B of Le Pucheuil, Therdonne and Bapaume Les Osiers.

The paleoanthropological level IIa of Biache-Saint-Vaast has recently been dated by means of ESR/U-series to 230 ± 24 ka BP [35] and could correspond to the beginning of MIS 7, which is consistent with the paleosoil overlying the sequence [35] and the zooarcheological analyses [36].

During MIS 7 (Figure 1B), Levallois technology also appeared in central Europe in levels 18–20 of Achenheim and Ariendorf 1 and endured in southern France at Gran Rois, level 1 of Petit Bost, unit 4 of Cantalouette I, level 4 of Barbas and Campsas, and on the Iberian Peninsula at Torralba and in level 2B of Galeria Pesada. In eastern Europe, the Levallois appeared in level Vb of Korolevo. At this chronological stage, evolved Acheulean technology has been found to coexist with the Levallois in northern Europe only at Bapaume Les Osiers, although such coexistence is predominant at lower latitudes.

During MIS 6 (Figure 1C), Levallois technology persisted in northern-central Europe at Le Cotte Saint Brelade, levels 15–17a of Achenheim and Markkleeberg. In southern France, the Levallois method endured in level 51 of Suard rockshelter, unit 4 of Coudoulous I, level 3 of Barbas, levels VIII-VII of Vaufrey Cave, unit IV of Baume Bonne and complex C of Lazaret Cave. Meanwhile, in the western Mediterranean, it reappeared on the Iberian Peninsula at level VII of Lezetxiki Cave, Cariguela Cave and Solana del Zamborino. At the end of MIS 6 the use of the Levallois method appeared in Croatia in level 1 of Krapina and in Italy in level 8 of trench F of Scario Cave. Evolved Acheulean industry is present only in complex C of Lazaret Cave.

### Review of the western Mediterranean archaeological record

On the Italian Peninsula the emergence of the Levallois is associated with the evolved Acheulean sites of Olio quarry and Rosaneto. At Olio quarry (Bologna) a large lithic assemblage was recovered when excavating the gravel deposits of the Indice River. A recent study recognized the presence of Levallois methods in the recurrent unidirectional convergent and recurrent bidirectional modalities [29]. The paleosoil associated with this discovery has been correlated with the Monte Mulino unit at the base of the San Mamante lithostratigraphy and with MIS 9 without any independent age tests [29]. The San Mamante section was discovered in 1988 during agricultural activities and was subsequently destroyed. Many of the paleosoils identified in the section were not found in other neighboring areas of the facing alluvial Po deposits [37]. Today, the fluvial terraces near Bologna have been clearly correlated with the alluvial stratigraphy of the Po plain and dated [38]–[40]. However, the destruction of the San Mamante section impeded the revision of the geological strata and the resolution of the problem of its chronology. In fact, in this lithostratigraphy, loess deposits are associated with MIS 6 [41]. The results of TL dates on archaeological flint discovered in loess sediments at the Apennine sites of Ghiardo and Ghiardello indicate instead an age of MIS 4 [42] in complete agreement with the dates of other loess deposits located in northern Italy, such as at the Bagaggera site [43] and in the Sorda Valley [44]. The chronological discrepancies in the San Mamante section have led the Olio quarry collection to be associated with a younger age.

At Rosaneto (Cosenza), an open-air site situated on a marine terrace at 60 meters above sea level, industry attributed to the evolved Acheulean due to the presence of choppers, handaxes and Levallois flakes was gathered on the red gravelly sand formation [45]. Malatesta and Zarlenga [46] associated the Rosaneto terrace with “Second Middle Pleistocene cycle deposits” without directly dating the sand. More recent geomorphological and stratigraphic research dated the second order terraces of Rosaneto (50–65 m above sea level) to the early Middle Pleistocene (800–650 ka BP) [47]. The absence of any archaeological excavation of the deposits or recovery of artifacts embedded in secure stratigraphic positions suggests that the assemblage should be considered palimpsests of different ages and that it has been incorrectly attributed to MIS 9.

The lithic assemblages of Olio quarry and Rosaneto have therefore been excluded from this debate due to their uncertain chronology. Another clarification concerns other sites that are sometimes associated with the early use of the Levallois method. At Colombo Cave (Savona, Italy), early Middle Paleolithic lithic assemblages were documented between levels 11 and 5 after the recovery of 13 atypical Levallois flakes [48]. A second cycle of research revealed that Levallois technology is inexistent [49] whereas recently another study point out again the typological description of some Levallois flakes [50]. The absence from level 5 to level 10 of Levallois cores and diagnostic pieces, such as trimming core flakes and predetermining Levallois flakes suggest that those blanks are actually products of discoid technology. At Torre in Pietra level d the lithic assemblage has been interpreted as Levallois [51] after a broadening of the criteria of Boeda [52]. The chronological attribution of this level has been largely debated and related firstly to the beginning of MIS5 on the base of geological correlations with the marine transgressions [53], [54] and successively to MIS7 [55] and to MIS7 or MIS6 [56] after the bio-chronological association of the faunal remains. The absence of direct dates of level d maintains uncertain its chronology that could cover a large temporal interval from MIS7 to MIS6. In unit III of Cueva Negra del Estrecho del Rio Quipar (Murcia, Spain) [57], what have been classified as Levallois flakes feature very obtuse butt angles and are more likely byproducts of centripetal flaking sequences. The artifacts associated with the Mousterian tradition due to steep, abrupt retouch are not very diagnostically useful and are in fact quite common in Paleolithic periods. At Bolomor Cave (Valencia, Spain), some authors have associated the lower assemblages with the early Middle Paleolithic due to the presence of scrapers and the typological categorization of Charentian Mousterian [58], [59], but Levallois technology is absent [60].

## Results

**Figure 2:**
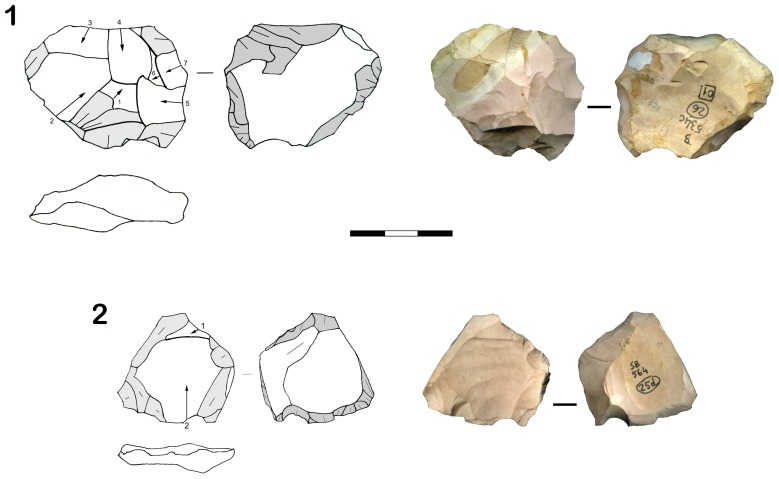
Lithic industry of lower stratigraphic units of San Bernardino Cave (Vicenza, Italy). Levallois recurrent centripetal core of unit VIII (1) and Levallois preferential core of unit VII (2).

Grotta Maggiore di San Bernardino (Vicenza) is located in northeastern Italy (45°06′N, 11°06′E). The cave is situated on the eastern slope of the Berici karst plateau, at 135 m above sea level facing the alluvial plain of the Bacchiglione River and the southern areas of the Euganei Hills (see Text S1, Figure S1). The stratigraphic sequence of San Bernardino Cave is made up of eight lithological units, and three main paleoclimatic cycles have been identified [61] (Figure S2). The lithic assemblage of unit VIII is composed of 470 flakes, 43 retouched tools and 22 cores, while 230 flakes, 29 retouched tools and 19 cores were recovered from unit VII (Table S3, S4, S5, S6). The technological analyses identified 1 Levallois recurrent centripetal core (Figure 2), 2 Levallois recurrent unidirectional flakes and 31 Levallois recurrent centripetal flakes in unit VIII (Figure S3, S4, S5). Unit VII yielded 1 Levallois preferential core (Figure 2), 11 Levallois recurrent unidirectional and 5 Levallois recurrent centripetal artifacts (see SI text, Figure S6, S7, S8). Within the Levallois technology identified, the centripetal flaking method was also detected in lesser percentages, in secondary *chaînes opératoires* such as unipolar and Kombewa.

### Chronology of the San Bernardino infill

**Figure 3:**
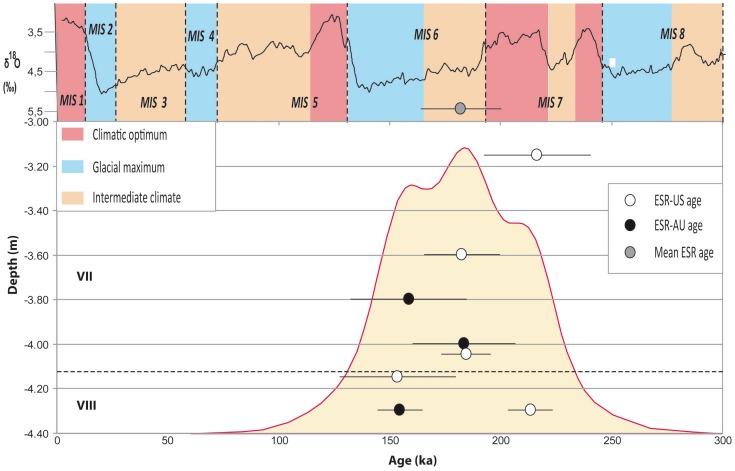
ESR/U-series ages of bones and teeth from the lower units VIII and VII of San Bernardino Cave (Vicenza, Italy). The ages are indicated with 1-σ errors.

Five bones from unit VII and two bones and one tooth from unit VIII were analyzed using the ESR-US technique [62]. As reported in various studies, bones often show evidence of uranium leaching. Because the determination of the p-parameter is not possible, a value equal to –1 is attributed to this parameter to calculate age. This procedure leads to an apparent systematic underestimation of ESR-US ages when the results are compared with other samples dated by other paleodosimetric methods such as luminescence (see [63]. A new uranium uptake model called the accelerated uptake model (AU), which combines incorporation followed by a leaching process, allows “minimum” ages to be determined in samples which have lost uranium after burial [64]. This is the case for most of the samples recovered from San Bernardino. The lower units of the stratigraphic sequence were found to have a minimum age range of between 154 and 214 ka (Table S1, S2). Unit VIII is comprised of gravelly anthropic layers with a marked presence of cervid remains, suggesting an interstadial period. Unit VII is made up of cryoclastic breccia embedded in loessic matrix, suggesting colder phases. Considering these data, the lower archaeological units of San Bernardino (units VIII and VII) may be contemporaneous with the extreme end of MIS 7 and the beginning of MIS 6 as shown by the mean age in Figure 3.

## Discussion

The new radiometric chronology of the lower units of San Bernardino Cave indicates that the introduction of the Levallois method in Europe occurred by means of diverse geographical patterns. This chronological review shows that the application of the Levallois method remained stable in northern Europe and southern France between the end of MIS 9 and MIS 6. In Spain, the presence of the Levallois is discontinuous with chronological gaps between MIS 9 and the end of MIS 7. In this fragmented scenario, the Italian Peninsula remained separate for a long temporal interval compared to other European territories, as the earliest secure evidence of Levallois production has been documented at the end of MIS 7 at San Bernardino Cave. The isolation of Italy from this technological advance has major evolutionary implications when the archaeological data are examined in conjunction with the paleoanthropological record.

The southern regions are considered glacial refugia during the Pleistocene, and were of considerable importance in the floral, faunal and human re-colonization of northern-central Europe after ice or cooling events [65], [66]. Although the Mediterranean territories underwent moderate environmental changes during the Middle Pleistocene and the mammalian community remained similar on the Iberian and Italian Peninsulas [67], these glacial refugia probably played different roles in faunal dispersal and hominin interaction because of their geographic and orographic diversity [68].

The analyses of the skeletal proportions and dental morphologies of Atapuerca fossils and their comparison with other contemporaneous hominins have demonstrated that the Sima de los Huesos (SH) population has greatly contributed to the body of knowledge on the biological development of Neanderthals [69]–[71]. These results first of all suggest diverse geographical displacements and interactions between SH groups and the population of northern-central Europe [72] and secondly assume the temporal coexistence of diverse hominin lineages [73], [74].

In Italy the human remains of the late Middle Pleistocene display strong archaic features [75]. A mandible and several teeth discovered at Visogliano (Trieste) (late MIS 11) are metrically comparable to ancient specimens such as *Homo erectus* [76]. The Ceprano calvarium, dated to 353 ± 4 ka BP (MIS 10) [77], lack any Neanderthal traits [75]. Moreover, cranial and post-cranial elements of Castel di Guido (end of MIS 9) maintained a prevalence of “erectus-like” features [75]. The geographical barriers of the Alps and the seas might have contributed to the inhabitants of the Italian Peninsula’s isolation from the genetic flow and interbreeding with other European populations.

In northern Europe the sudden increase in the use of the Levallois is chronologically related to the biological development towards Neanderthal speciation that most likely took place during MIS 8 [78], [79] and was established at the beginning of MIS 7, as demonstrated by the braincase morphology of Biache 1 [80]. Meanwhile, on the Italian Peninsula the fragmentary collections of fossils dated to around 300–250 ka, such as the parietal bone from Casal de’ Pazzi, the femur and metatarsal from Sedia del Diavolo, the femoral shaft from Ponte Mammolo and the undated human remains of Lamalunga Cave (Altamura), still display a mixture of archaic morphologies and progressive features [75]. Although these fossil specimens are interpreted as “transitional”, the speciation event towards *Homo neanderthalensis* is postdated to MIS 6, as demonstrated by the crania of the two early Neanderthals from Saccopastore (MIS 5) [81]. The evidence that the introduction of the Levallois and these speciation events correspond both chronologically and geographically is striking.

From a technological point of view, the lithic series of the lower units of San Bernardino are consistent with the general pattern of the emergence of the Levallois in the western Mediterranean, while the lithic assemblages of Monte delle Gioie, Sedia del Diavolo and Casal de’ Pazzi document the persistence of chopping tools associated with centripetal and bipolar technology [82]. In fact, in northern Europe at the bridge between MIS 9 and MIS 8, the “proto”-Levallois technologies of Purfleet, Mesvin IV and Kesselt-Op de Schans were characterized by the simple preparation of the striking platform [83]. This early stage lacked the unidirectional development of hierarchized technologies [84] that characterized the later Levallois assemblages in this area [18], [83]. In southern France and in the western Mediterranean, the adoption of hierarchized technologies passed through the elaboration of centripetal methods and coexisted with handaxe shaping. Only after this initial step did the preferential and recurrent unidirectional exploitation of cores emerge. The only exception is found at the Les Bosses site, in which all of the Levallois modalities described by Boëda [52] were identified. This trend of the southern European territories is also documented at San Bernardino Cave in which the technological readings of the lithic series confirm the shift from the recurrent centripetal modalities of unit VIII (MIS 7a) to the recurrent unidirectional of unit VII (MIS 6), although the industries are complemented only by unhierarchical reductions lacking any handaxe production (see SI Text).

The other important difference concerns the final products pursued through the use of Levallois reduction sequences. In northern Europe, at the bridge between MIS 9 and MIS 8 as well as between MIS 8 and MIS 7, the adoption of the Levallois method is related to the systematic production of points that were occasionally retouched and thinned on the proximal end, probably to facilitate hafting with wooden implements [23], [83], [85], [86]. Meanwhile, in southern Europe Levallois technology was used in the production of large and more standardized flakes and Levallois points were produced in lesser percentages. Again, a similar pattern is seen in the lower units of San Bernardino. Levallois blanks are numerous in the assemblages and stone tools are represented mainly by scrapers and denticulates (Table S6).

These macro-regional similarities suggest that the adoption of analogous Levallois recurrent modalities might not be the result of technological convergence due to the natural properties of flaked stones, but rather to similar cognitive advances and somehow interconnected developments in technical behavior. This pattern might have been facilitated by “culturally mediated migration”, a mechanism by which an individual can migrate to groups that surpass a given level of cultural familiarity [87]. Social studies demonstrate that small groups tend to be less receptive to the introduction of novelties [88] and that once an innovation has been locally accepted the spread to neighboring territories might meet with more resistance by foreign groups. Although an increase in population density has been considered a critical factor in encouraging the adoption of innovation [89], another important social barrier is culture in the sense of way of life and technology. Thus the scattered introduction of the Levallois method and its coexistence after these speciation events with the Acheulean core flaking method might be considered in terms of cultural differentiations between distinct populations.

The introduction of the Levallois in the lower units of San Bernardino Cave significantly predates the adoption of this technology compared to the rest of the Italian territory and level d of Torre in Pietra [51] might be contemporaneous or posterior. Considerable evidence has been documented in level 8 of Scario Cave [90], dated to the end of MIS 6, and during the MIS 5 in level 10 of Barma Grande [91], at the open-air sites of Erbarella, Monte Conero, Colonia Montani and Ponte di Crispiero [27], [92], in level 6 of Scario Cave, level 17 of Poggio rockshelter [93] and at Taddeo Cave [27].

Although the Levallois was latent in Acheulean core flaking strategies, in addition to local development, possible contact between the populations on the Italian Peninsula and those from eastern-central Europe might also be considered. The molar tooth recovered from level 6 of Poggio Cave (pre-Eemian by means of stratigraphical correlation and associated with Acheulean industry) [94] and the infant mandible from level 51 of Molare rockshelter (attributed to MIS5 and associated with no Levallois industry) [95]–[97], considered “transitional” due to some archaic traits, present marked similarities to the Krapina samples. Moreover, the identification of a genetic mtDNA group in the western Mediterranean [98] does not reject the hypothesis of early Neanderthal displacements and the consequential expansion of technological innovations.

## Conclusion

The introduction of the Levallois method in Europe was an asynchronous event involving the reorganization of local core technology with chronological differences between European territories. The review of some Mediterranean sites and the new data from the lower units of San Bernardino Cave point to the concomitance of the rise of this new technology and major biological advances in the European population, which led to the speciation of Neanderthals [78], [79], [81]. The geographical differences in the early stages of Levallois developments might reflect not only economic patterns in the management of resources, but also regional differences in speciation events, as evidenced by the genetic variability [98] and body proportions of Neanderthal populations [99]. The new data from units VIII and VII of San Bernardino Cave raise the issue of the different roles of glacial refugia in the peopling and spread of new technologies across Europe during the late Middle Pleistocene.

## Materials and Methods

### Ethics Statement

All necessary permits were obtained from the Italian Ministery of Culture for the described study, which complied with all relevant regulations. The identification numbers of the specimens analysed range from IG5647 to IG5698.

Repository information: the specimen is temporary housed at the University of Ferrara, in the Section of Prehistory and Anthropology, Corso Ercole I d’Este Ferrara, Italy, with the permission of the Ministry of Culture - Veneto Archaeological Superintendence.

The ESR-US method uses principles of radiation dosimetry to determine the time since an object’s last exposure to light and the formation of minerals. ESR signals change with the natural ionizing radiation dose, and thus with time. The equivalent dose (DE) and the dose rate (annual dose) are determined in a series of analytical steps. The age is then obtained from the ratio of these two measures. Different types of material, like mammal teeth or bones, can be dated when they are buried, as in the case of San Bernardino. Important European sites, such as La Micoque, Baume Bonne, Biache and Arago in France, Gran Dolina and Ambrona in Spain, Visogliano and Isernia la Pineta in Italy have already been dated by means of this method.

## Supporting Information

Figure S1 **Geographical map of the North-East of Italy with position of San Bernardino Cave (1) in the Berici Hills.**
(JPG)

Figure S2 **The stratigraphy of the San Bernardino Cave from units VIII to II.** Key: 1. disturbed deposit with medieval finds; 2. bioturbation; 3. main palaeo-living floors; 4. loess; 5. thermoclastic breccia; 6. limestone gravel; 7. paleosoil.
(JPG)

Figure S3 **Levallois Recurrent Centripetal flakes (1-7), refitting centripetal flakes (8) of Unit VIII.**
(TIF)

Figure S4 **Centripetal cores (1-3) and core-on-flakes (4-5) of Unit VIII.** The scar orientation is indicated by the arrow, a black dot indicates the presence of a negative bulb, and the grey color indicates the presence of cortex.
(TIF)

Figure S5 **Retouched tools of Unit VIII.**
(TIF)

Figure S6 **Levallois Recurrent Unidirectional flakes (1-2),** Levallois Recurrent Centripetal flakes (3-5), refitting core-edge flake (6) of Unit VII.
(TIF)

Figure S7 **Centripetal (1-2), unidirectional (3) and laminar (4) cores of Unit VII.**
(TIF)

Figure S8 **Retouched tools of Unit VII.**
(TIF)

Table S1 **Uranium content, isotopic ratios, initial bone and enamel thickness, and removed enamel or external part of bones T allowing the elimination of external alpha contribution in the annual dose rate calculation and Equivalent doses of San Bernardino samples.**
(DOC)

Table S2 **Annual dose rate, p or n values according the US or AU model used respectively and age of bones and tooth of San Bernardino site.**
(DOC)

Table S3 **Total number of the lithic assemblages of Units VIII and VII.**
(DOC)

Table S4 **Raw counts and percentages of knapping products of Units VIII and VII.**
(DOC)

Table S5 **Raw counts and percentages of cores of Units VIII and VII.**
(DOC)

Table S6 **Raw counts and percentages of retouched tools of Unit VIII and VII.**
(DOC)

Data Set S1 (DOC)

Text S1 **Archaeological Context.**
(DOC)
