# Supplementary figures and images for: San Bernardino Cave (Italy) and the Appearance of Levallois Technology in Europe: Results of a Radiometric and Technological Reassessment
Source: PLoS One. 2013 Oct 16;8(10):e76182. doi: 10.1371/journal.pone.0076182 (PMC3797834; doi:10.1371/journal.pone.0076182)

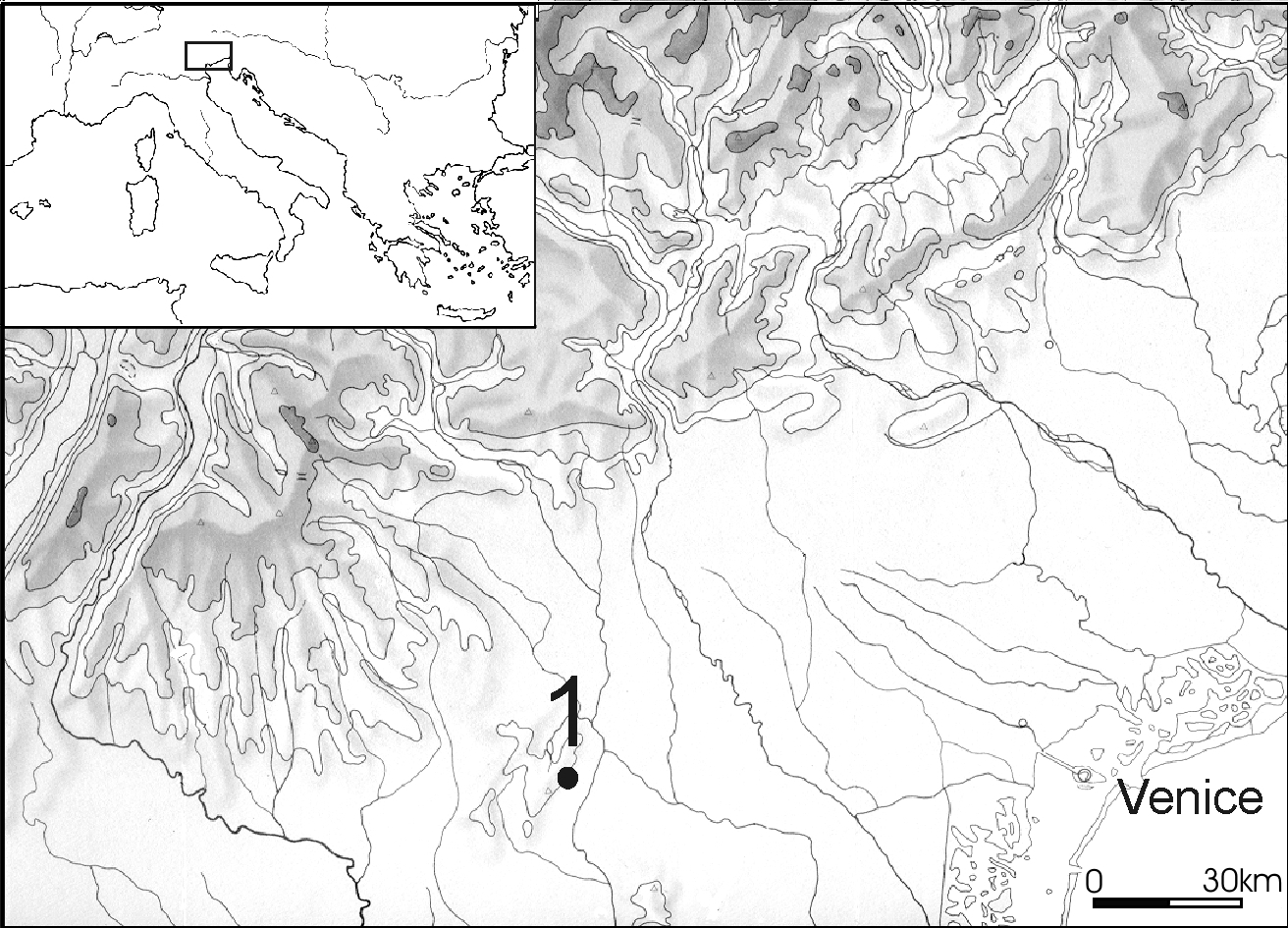

Supplement: Figure S1 — Geographical map of the North-East of Italy with position of San Bernardino Cave (1) in the Berici Hills. (JPG) [file pone.0076182.s001.jpg]

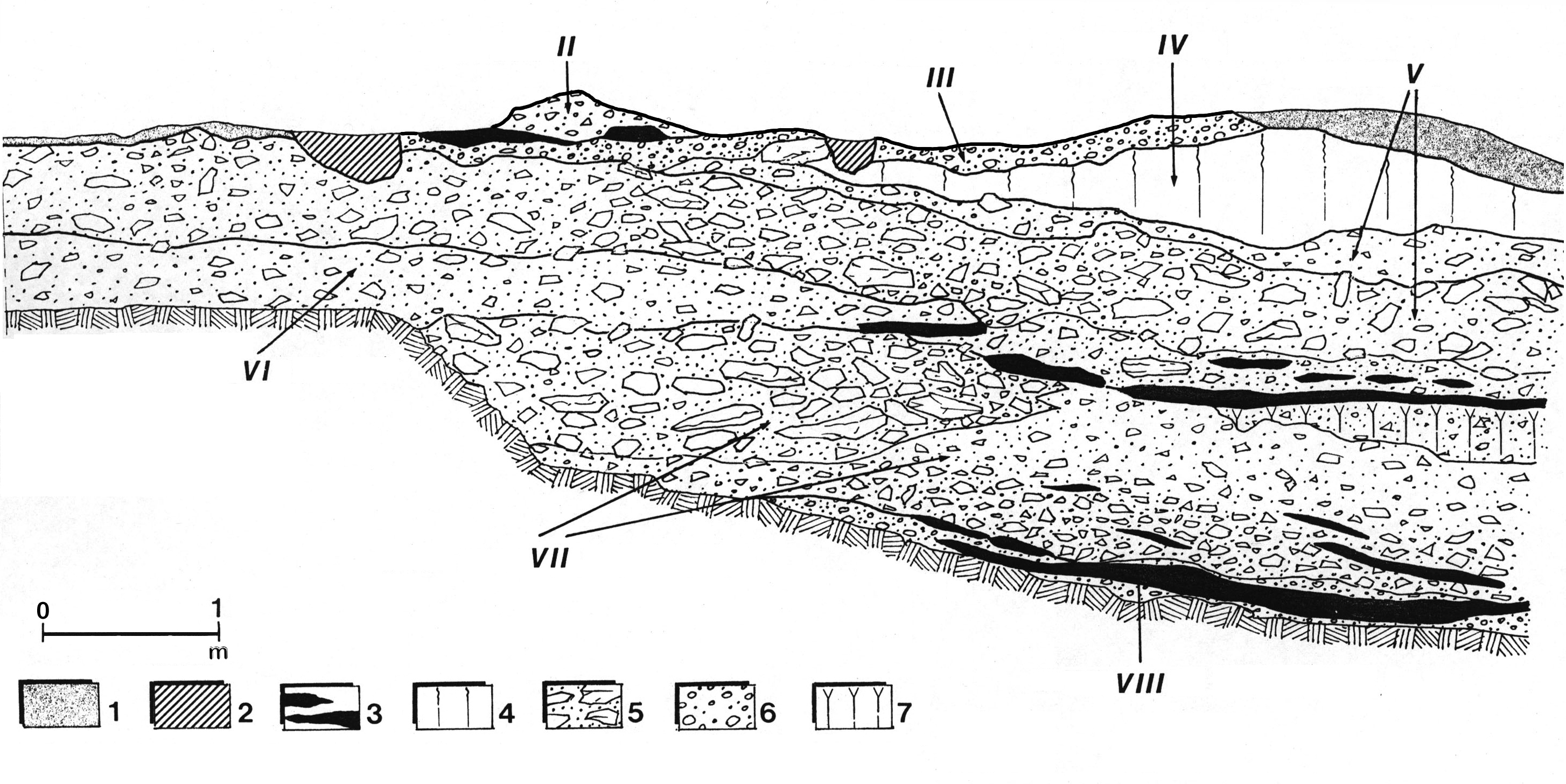

Supplement: Figure S2 — The stratigraphy of the San Bernardino Cave from units VIII to II. Key: 1. disturbed deposit with medieval finds; 2. bioturbation; 3. main palaeo-living floors; 4. loess; 5. thermoclastic breccia; 6. limestone gravel; 7. paleosoil. (JPG) [file pone.0076182.s002.jpg]

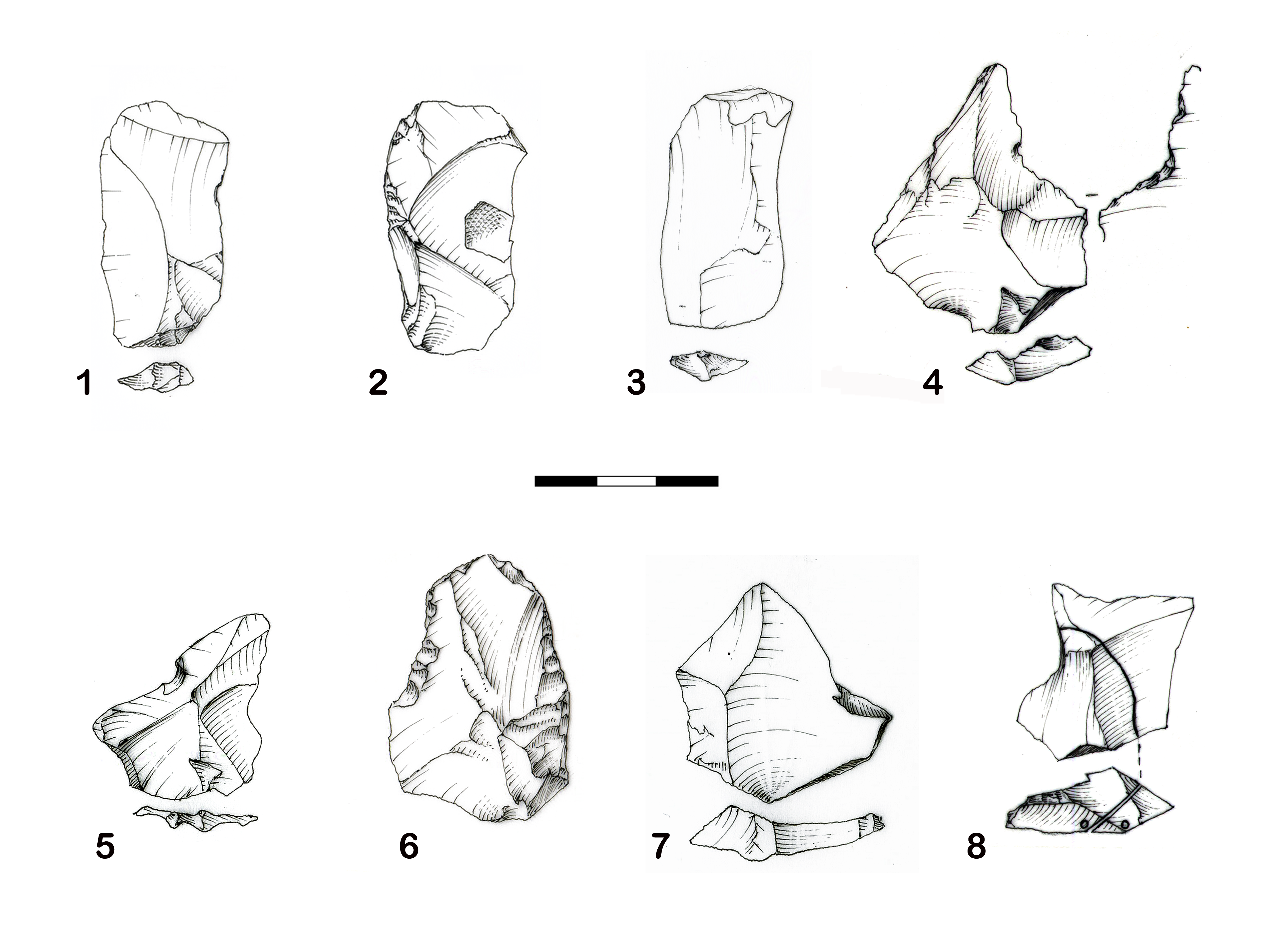

Supplement: Figure S3 — Levallois Recurrent Centripetal flakes (1-7), refitting centripetal flakes (8) of Unit VIII. (TIF) [file pone.0076182.s003.tif]

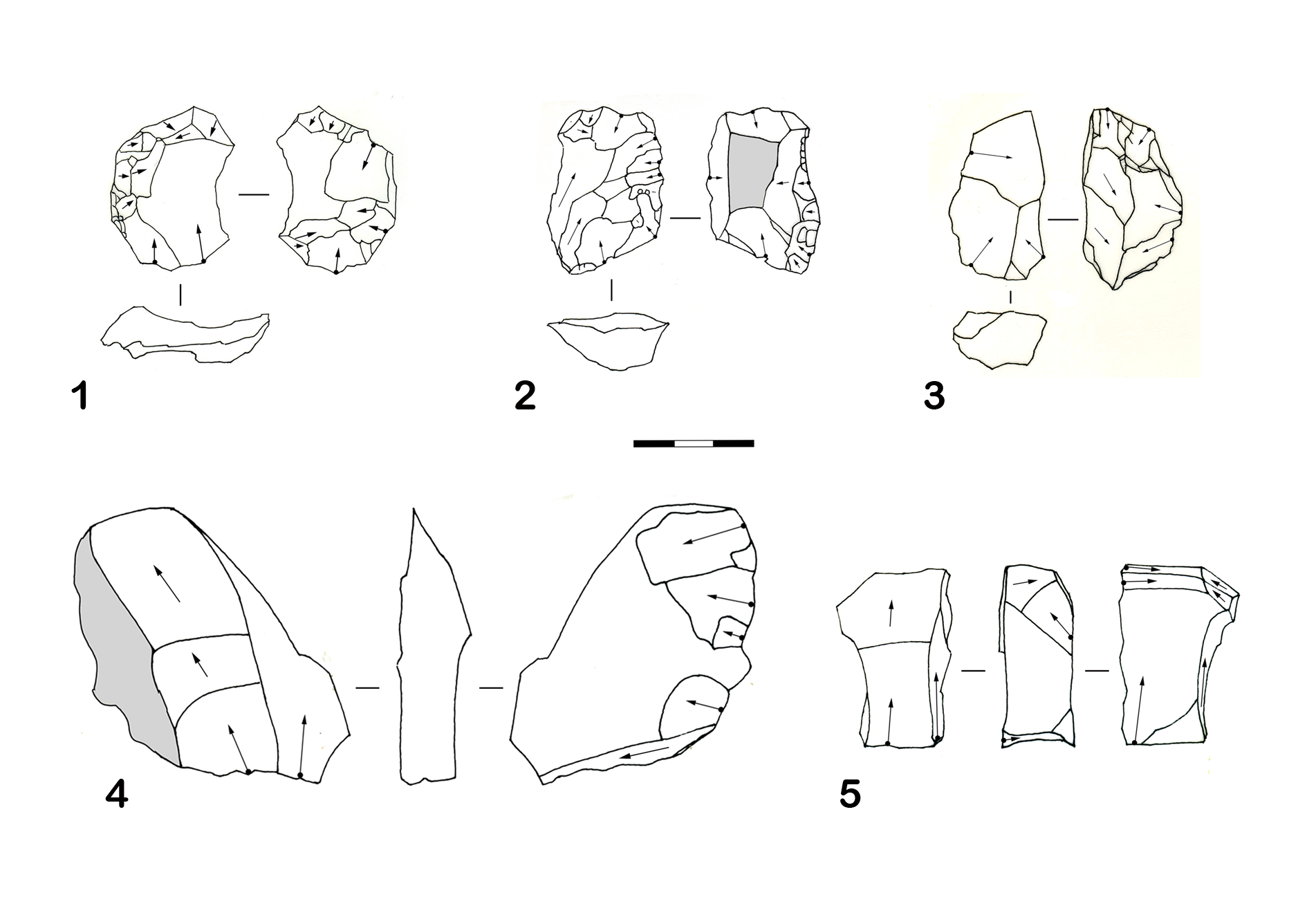

Supplement: Figure S4 — Centripetal cores (1-3) and core-on-flakes (4-5) of Unit VIII. The scar orientation is indicated by the arrow, a black dot indicates the presence of a negative bulb, and the grey color indicates the presence of cortex. (TIF) [file pone.0076182.s004.tif]

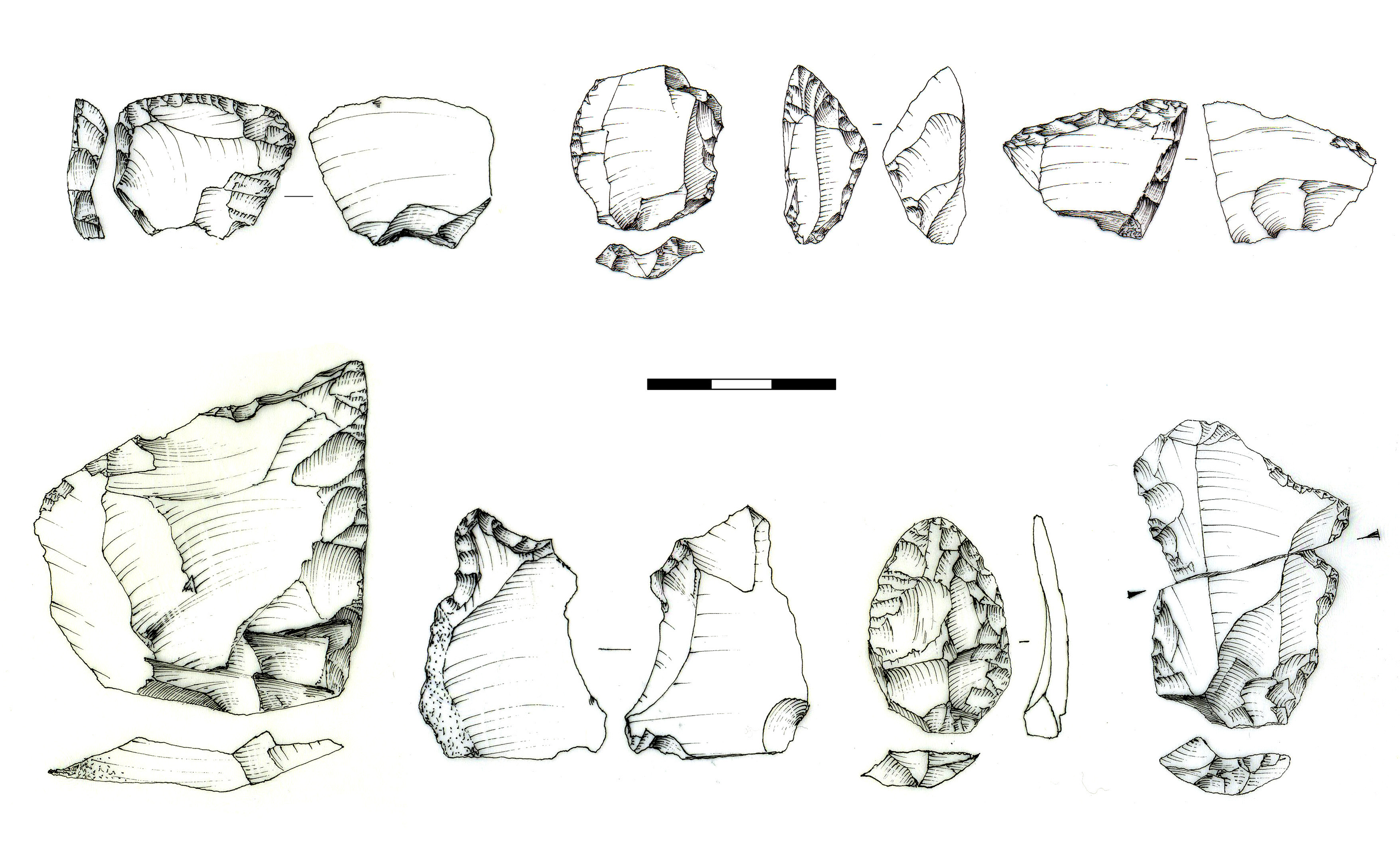

Supplement: Figure S5 — Retouched tools of Unit VIII. (TIF) [file pone.0076182.s005.tif]

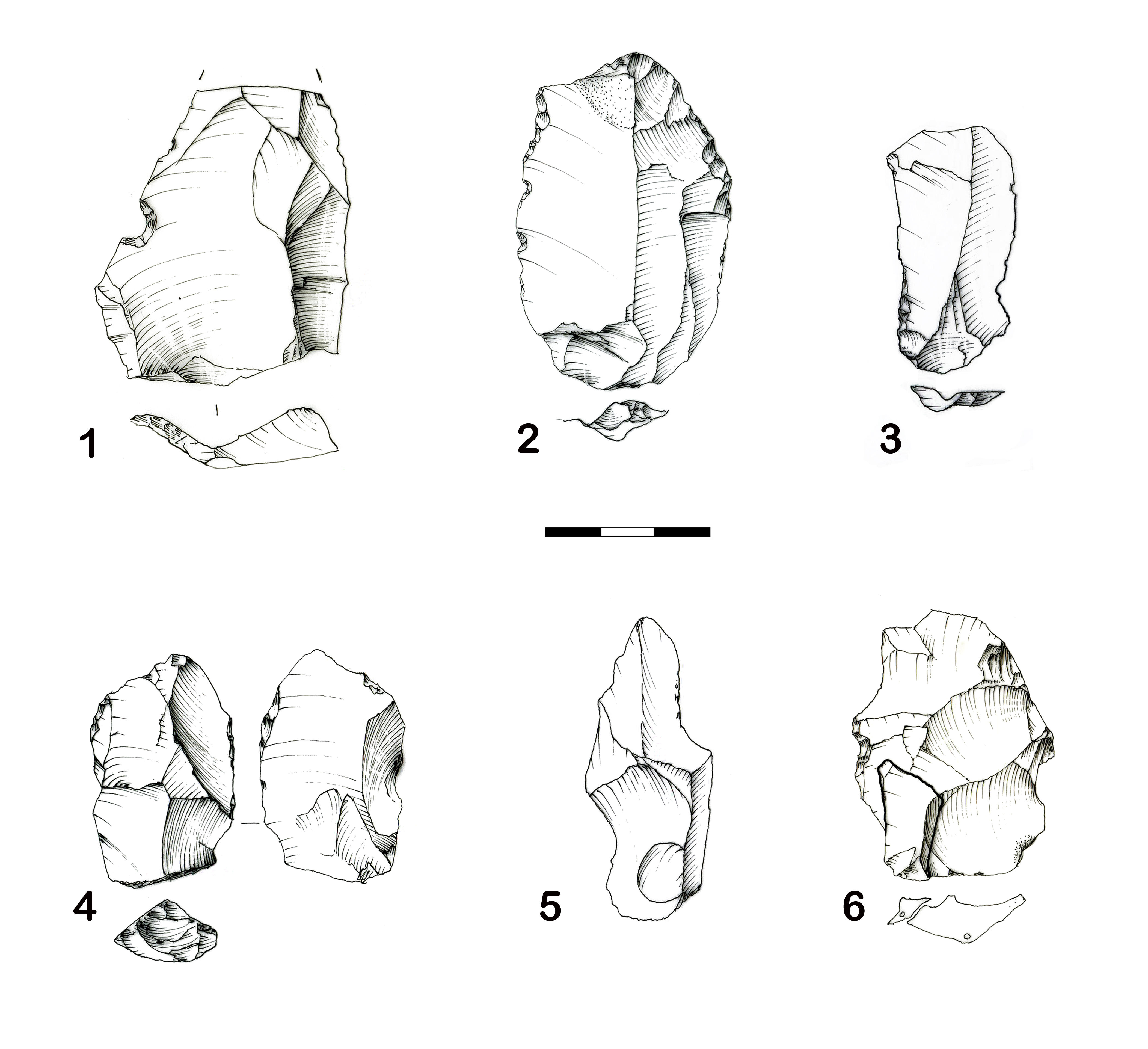

Supplement: Figure S6 — Levallois Recurrent Unidirectional flakes (1-2), Levallois Recurrent Centripetal flakes (3-5), refitting core-edge flake (6) of Unit VII. (TIF) [file pone.0076182.s006.tif]

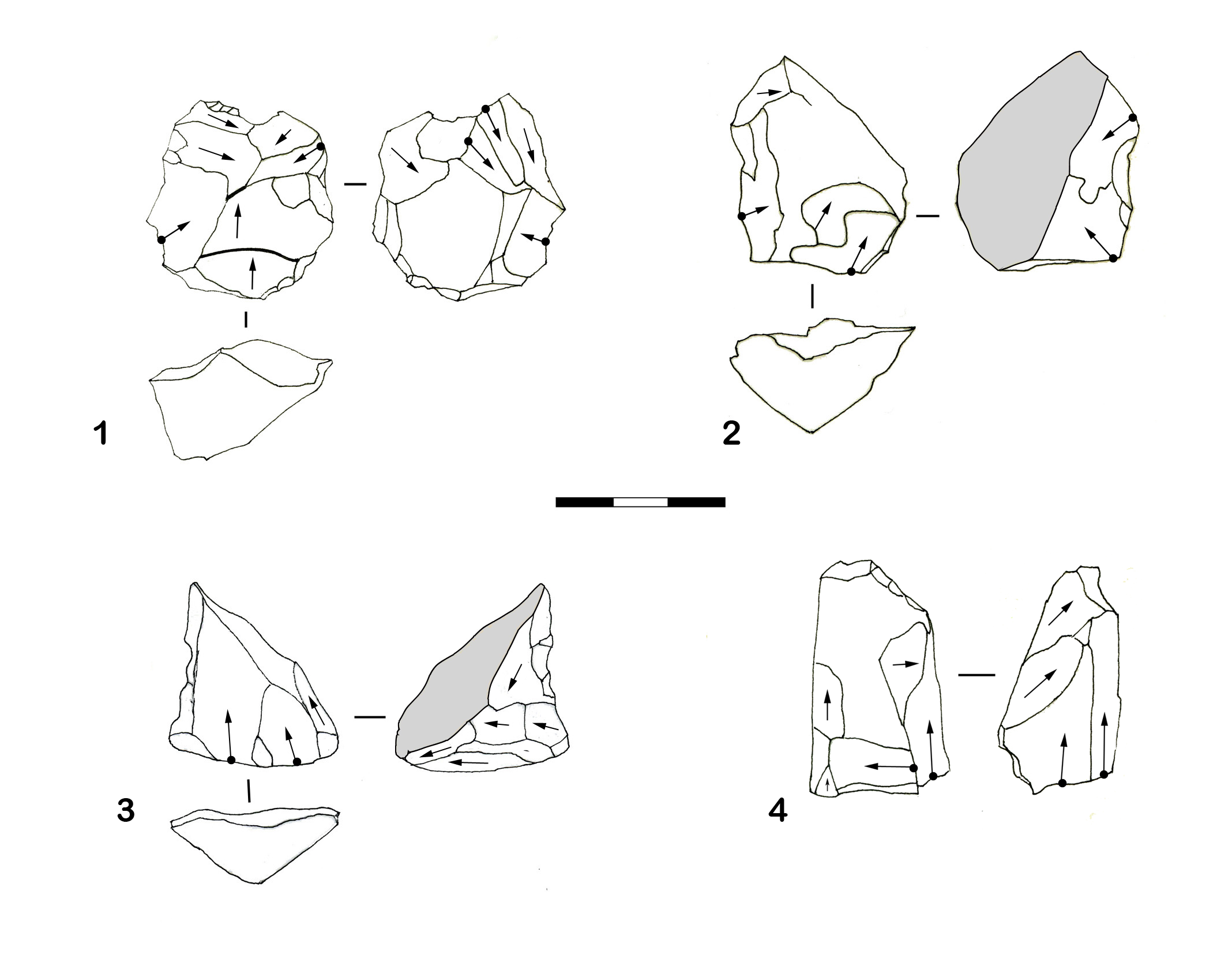

Supplement: Figure S7 — Centripetal (1-2), unidirectional (3) and laminar (4) cores of Unit VII. (TIF) [file pone.0076182.s007.tif]

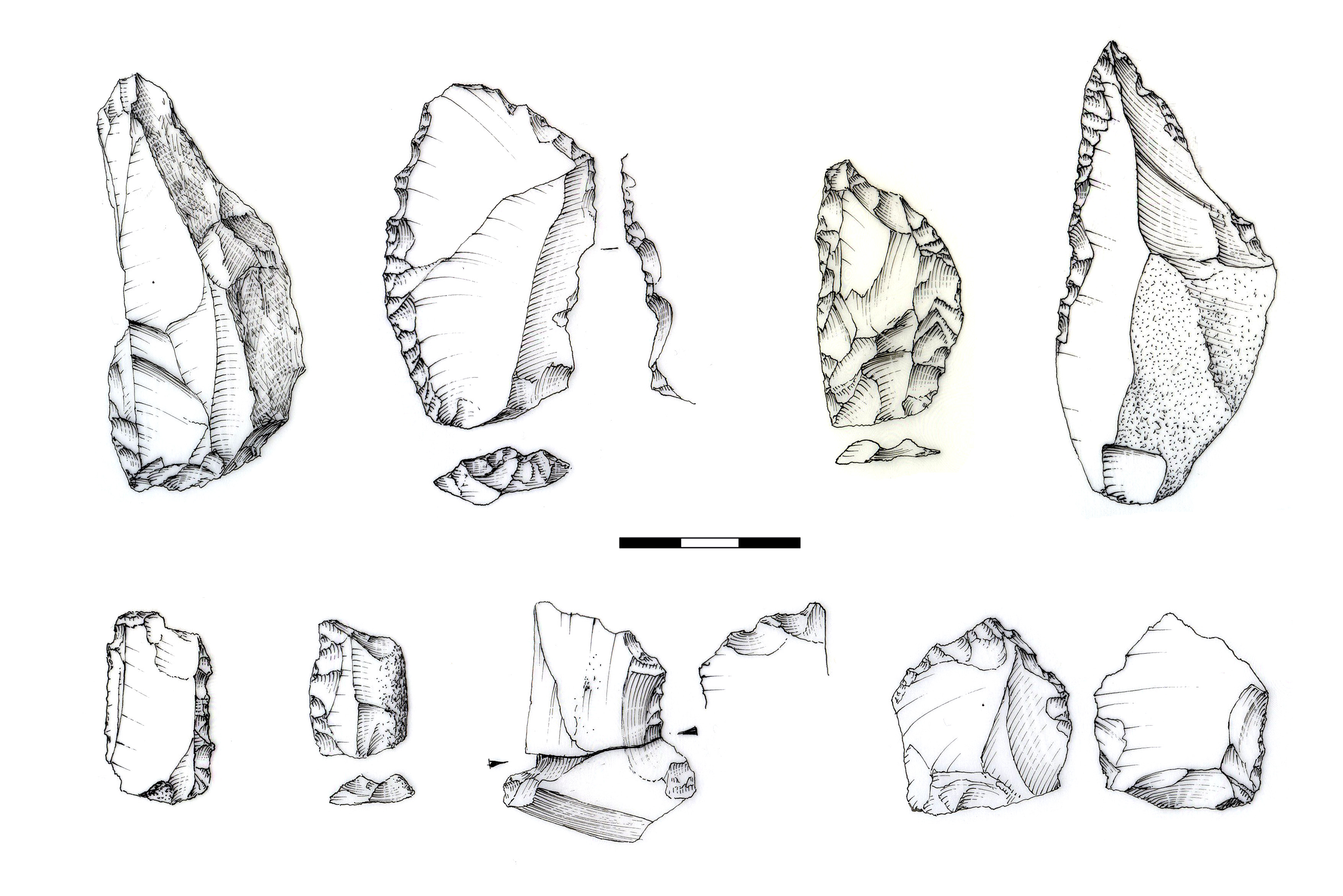

Supplement: Figure S8 — Retouched tools of Unit VII. (TIF) [file pone.0076182.s008.tif]
