# Supplementary material for: San Bernardino Cave (Italy) and the Appearance of Levallois Technology in Europe: Results of a Radiometric and Technological Reassessment
Source: PLoS One. 2013 Oct 16;8(10):e76182. doi: 10.1371/journal.pone.0076182 (PMC3797834; doi:10.1371/journal.pone.0076182)
Supplement: Table S1 — Uranium content, isotopic ratios, initial bone and enamel thickness, and removed enamel or external part of bones T allowing the elimination of external alpha contribution in the annual dose rate calculation and Equivalent doses of San Bernardino samples. (DOC) [file pone.0076182.s009.doc]

| Sample | Tissue | U (ppm) | 234U/238U | 230Th/234U | 222Rn/230Th | T enamel (μm) | removed T * (μm) | DE (Gy) |
| --- | --- | --- | --- | --- | --- | --- | --- | --- |
| SB27 | bone | 9,33 | 1.045 ± 0.010 | 0.856 ± 0.031 | 0,578 | 3375 | 297 | 165 ± 6 |
|  |  |  |  |  |  |  |  |  |
| SB28 | bone | 12,75 | 1.032 ± 0.022 | 0.749 ± 0.039 | 0,36 | 9534 | 297 | 164 ± 7 |
|  |  |  |  |  |  |  |  |  |
| SB30 | bone | 10,65 | 1.108 ± 0.025 | 0.868 ± 0.023 | 0,373 | 4219 | 297 | 140 ± 10 |
|  |  |  |  |  |  |  |  |  |
| SB32 | bone | 11,316 | 1.059 ± 0.031 | 0.859 ± 0.030 | 0,506 | 8944 | 297 | 170 ± 8 |
|  |  |  |  |  |  |  |  |  |
| SB33 | bone | 14,1 | 1.039 ± 0.024 | 0.830 ± 0.025 | 0,421 | 8438 | 297 | 206 ± 9 |
|  |  |  |  |  |  |  |  |  |
| SB34 | bone | 9,5 | 1.117 ± 0.018 | 1.131 ± 0.026 | 0,346 | 3375 | 297 | 144 ± 5 |
|  |  |  |  |  |  |  |  |  |
| SB35 | bone | 11,88 | 1.047 ± 0.030 | 0.858 ± 0.029 | 0,425 | 3969 | 297 | 219 ± 5 |
|  |  |  |  |  |  |  |  |  |
| SB0307 | enamel | 0,74 | 0.986 ± 0.126 | 1.123 ± 0.118 | 0,21 | 954 | 116 | 75.4 ± 3.8 |
|  | dentine | 16,38 | 0.933 ± 0.038 | 1.015 ± 0.052 |  |  | 88 |  |

Table S1: Uranium content, isotopic ratios, initial bone and enamel thickness, and removed enamel or external part of bones T allowing the elimination of external alpha contribution in the annual dose rate calculation and Equivalent doses of San Bernardino samples.
