# Supplementary material for: San Bernardino Cave (Italy) and the Appearance of Levallois Technology in Europe: Results of a Radiometric and Technological Reassessment
Source: PLoS One. 2013 Oct 16;8(10):e76182. doi: 10.1371/journal.pone.0076182 (PMC3797834; doi:10.1371/journal.pone.0076182)
Supplement: Table S2 — Annual dose rate, p or n values according the US or AU model used respectively and age of bones and tooth of San Bernardino site. (DOC) [file pone.0076182.s010.doc]

| Sample | Units | Layer | γ + cosmic | Internal dose rate | ß dose rate | Total dose rate | n-value | AU (or *US**) |
| --- | --- | --- | --- | --- | --- | --- | --- | --- |
|  |  |  | (μGy/a) | (μGy/a) | (μGy/a) | (μGy/a) | (or *p-value**) | Age (ka) |
|  |  |  |  |  |  |  |  |  |
|  | **VII** |  |  |  |  |  |  |  |
| SB27 |  | 25b | 175 ± 14 | 575 ± 87 | 12 ± 1 | 760 ± 88 | *-0.95 ± 0.05** | *217 ± 24* |
| SB28 |  | 25c | 168 ± 10 | 724 ± 91 | 4 ± 0 | 896 ± 92 | *-0.84 ± 0.06** | *183 ± 17* |
| SB30 |  | 25d | 163 ± 13 | 708 ± 156 | 10 ± 1 | 881 ± 157 | -0.0094 ± 0.0020 | 159 ± 26 |
| SB32 |  | 25e | 157 ± 12 | 763 ± 122 | 4 ± 0 | 924 ± 123 | -0.0078 ± 0.0013 | 184 ± 23 |
| SB33 |  | 25f | 156 ± 12 | 936 ± 82 | 4 ± 0 | 1114 ± 84 | *-0.97 ± 0.03** | *185 ± 11* |
|  | **VIII** |  |  |  |  |  |  |  |
| SB34 |  | 26a | 97 ± 9 | 830 ± 161 | 8 ± 1 | 935 ± 161 | -0.0101 ± 0.0067 | 154 ± 26 |
| SB35 |  | 26 | 235 ± 21 | 782 ± 49 | 6 ± 0 | 1023 ± 53 | *-0.96 ± 0.02** | *214 ± 10* |
| SB0307 |  | 26 bottom | 114 ± 12 | 196 ± 2 | 176 ± 5 | 486 ± 10 | -0.0108 ± 0.0007 (e) | 155 ± 10 |
|  |  |  |  |  |  |  | -0.0107 ± 0.0008 (d) |  |

Table S2: Annual dose rate, p or n values according the US or AU model used respectively and age of bones and tooth of San Bernardino site.
