# Supplementary material for: San Bernardino Cave (Italy) and the Appearance of Levallois Technology in Europe: Results of a Radiometric and Technological Reassessment
Source: PLoS One. 2013 Oct 16;8(10):e76182. doi: 10.1371/journal.pone.0076182 (PMC3797834; doi:10.1371/journal.pone.0076182)
Supplement: Table S3 — Total number of the lithic assemblages of Units VIII and VII. (DOC) [file pone.0076182.s011.doc]

| Unit VIII | | TYPE | Unit VII | |
| --- | --- | --- | --- | --- |
| Nº | *%* |  | Nº | % |
| 470 | *4.9* | Flakes | 230 | *5.6* |
| 769 | *8* | Fragments | 905 | *21.9* |
| 8281 | *86.3* | Debris | 2950 | *71.3* |
| 43 | *0.4* | Retouched tools | 29 | *0.7* |
| 22 | *0.2* | Cores | 19 | *0.5* |
| 11 | *0.1* | Cores fragments | 4 | *0.1* |
| 9596 | *100* | TOTAL | 4137 | *100* |

Table S3: Total number of the lithic assemblages of Unit VIII - VII.
