# Supplementary material for: San Bernardino Cave (Italy) and the Appearance of Levallois Technology in Europe: Results of a Radiometric and Technological Reassessment
Source: PLoS One. 2013 Oct 16;8(10):e76182. doi: 10.1371/journal.pone.0076182 (PMC3797834; doi:10.1371/journal.pone.0076182)
Supplement: Table S4 — Raw counts and percentages of knapping products of Units VIII and VII. (DOC) [file pone.0076182.s012.doc]

| Unit VIII | | TYPE | Unit VII | |
| --- | --- | --- | --- | --- |
| Nº | % |  | Nº | % |
| 34 | *7.2* | Cortex >50% | 13 | *5.7* |
| 50 | *10.6* | Cortex <50% | 36 | *15.7* |
| 25 | *5.3* | Naturally backed flakes | 16 | *7* |
| 71 | *15.1* | Trimming of striking platform | 14 | *6.1* |
| 73 | *15.5* | Predetermining Levallois flakes | 38 | *16.5* |
| 13 | *2.8* | Predetermined indet. flakes |  |  |
| 2 | *0.4* | Levallois recurrent unidirectional flakes | 11 | *4.8* |
| 31 | *6.6* | Levallois recurrent centripetal flakes | 5 | *2.2* |
| 14 | *3* | Pseudo-Levallois points | 4 | *1.7* |
| 35 | *7.4* | Core-edge removal flakes | 24 | *10.4* |
| 4 | *0.9* | Centripetal flakes | 7 | *3* |
| 2 | *0.4* | Orthogonal flakes |  |  |
| 9 | *1.9* | Laminar flakes | 8 | *3.5* |
| 6 | *1.3* | Unidirectional flakes | 8 | *3.5* |
| 13 | *2.8* | Kombewa-type flakes | 14 | *6.1* |
| 30 | *6.4* | Re-shaping of flaking surface | 4 | *1.7* |
| 58 | *12.3* | Knapping accident flakes | 28 | *12.2* |
| 470 | *100* | TOTAL | 231 | *100* |

Table S4: Raw counts and percentages of knapping products of Unit VIII-VII.
