# Supplementary material for: San Bernardino Cave (Italy) and the Appearance of Levallois Technology in Europe: Results of a Radiometric and Technological Reassessment
Source: PLoS One. 2013 Oct 16;8(10):e76182. doi: 10.1371/journal.pone.0076182 (PMC3797834; doi:10.1371/journal.pone.0076182)
Supplement: Table S5 — Raw counts and percentages of cores of Units VIII and VII. (DOC) [file pone.0076182.s013.doc]

| Unit VIII | | TYPE | Unit VII | |
| --- | --- | --- | --- | --- |
| Nº | % |  | Nº | % |
|  |  | Levallois preferential cores | 1 | *4.3* |
| 1 | *3* | Levallois recurrent centripetal cores |  |  |
| 7 | *21.2* | Centripetal cores | 6 | *26.1* |
|  |  | Unidirectional cores | 6 | *26.1* |
|  |  | Laminar core | 1 | *4.3* |
| 1 | *3* | Polyhedral cores |  |  |
| 13 | *39.4* | Core-on-flakes | 5 | *21.7* |
| 11 | 33.3 | Cores fragments | 4 | 17.4 |
| 33 | 100 | TOTAL | 23 | *100* |

Table S5: Raw counts and percentages of cores of Unit VIII and VII.
