# Supplementary material for: San Bernardino Cave (Italy) and the Appearance of Levallois Technology in Europe: Results of a Radiometric and Technological Reassessment
Source: PLoS One. 2013 Oct 16;8(10):e76182. doi: 10.1371/journal.pone.0076182 (PMC3797834; doi:10.1371/journal.pone.0076182)
Supplement: Table S6 — Raw counts and percentages of retouched tools of Unit VIII and VII. (DOC) [file pone.0076182.s014.doc]

| Unit VIII | | TYPE | Unit VII | |
| --- | --- | --- | --- | --- |
| Nº | % |  | Nº | % |
| 25 | *58.1* | Scrapers | 15 | *51.7* |
| 6 | *14* | Convergent Scrapers | 1 | *3.4* |
|  |  | Double Scrapers | 2 | *6.9* |
| 3 | 7 | Multiple Tools |  |  |
| 3 | *7* | Notched Tools | 4 | *13.8* |
| 6 | *14* | Denticulates | 7 | *24.1* |
| 43 | *100* | TOTAL | 29 | *100* |

Table S6: Raw counts and percentages of retouched tools of Unit VIII- VII.
