# Supplementary material for: San Bernardino Cave (Italy) and the Appearance of Levallois Technology in Europe: Results of a Radiometric and Technological Reassessment
Source: PLoS One. 2013 Oct 16;8(10):e76182. doi: 10.1371/journal.pone.0076182 (PMC3797834; doi:10.1371/journal.pone.0076182)
Supplement: Data Set S1 — (DOC) [file pone.0076182.s015.doc]

| **SITE** | **LEVEL** | **LOCATION** | **MIS** | **DATE (ka BP)** | **METHOD** | **REFERENCE** |
| --- | --- | --- | --- | --- | --- | --- |
| Purfleet |  | UK | late MIS 9 | 324 | TL |  |
| Mesvin IV |  | Belgium | early MIS 8 | 275 +38/-29 | ^230^Th/^234^U |  |
|  |  |  |  | 298 + 50/-35 |  |  |
| Kesselt-Op de Schans |  | Belgium | early MIS 8 | 300 | Geology |  |
| Markkleeberg |  | Germany | early MIS 8 | 240-300 | Geology |  |
| Orgnac 3 | level 2 | France | early MIS 8 | 298 ± 55 | U series |  |
|  | level 5b |  | early MIS 8 | 288 +82/- 45 | ^230^Th/^234^U |  |
|  | level 6 |  | MIS 11 | 374 +165/- 94 |  |  |
|  |  |  | MIS 9 | 309 ± 34 | ESR |  |
| Les Bosses | level 1 | France | late MIS 9/ early MIS 8 | 217 ± 24 | TL |  |
|  |  |  |  | 258 ± 21 |  |  |
|  |  |  |  | 280 ± 31 |  |  |
|  |  |  |  | 320 ± 38 |  |  |
|  |  |  |  | 328 ± 32 |  |  |
|  |  |  |  | 345 ± 41 |  |  |
| La Micoque | level L 2/3 | France | MIS 8-9 | 288 - 350 | ESR/U series |  |
| Raspide 2 |  | France | MIS 8 |  | Geology |  |
| Petit Bost | level 2 | France | MIS 9 | 312 ± 43 | TL |  |
|  |  |  | MIS 10 | 338 ± 43 |  |  |
| Gran Dolina | unit TD11 | Spain | MIS 7 | 240 ± 44 | TL/IRSL |  |
|  | unit TD10.2 |  | MIS 7 | 244 ± 26 |  |  |
|  |  |  | MIS 10 | 430 ± 39 |  |  |
|  | unit TD10.1 |  | MIS 8 | 308 ± 46 | ESR/U series |  |
|  |  |  | MIS 8 | 332 ± 50 |  |  |
|  |  |  | MIS 11 | 390 ± 59 |  |  |
|  |  |  | MIS 10 | 379 ± 57 |  |  |
|  | unit TD10.2 |  | MIS 9 | 337 ± 51 |  |  |
|  |  |  | MIS 10 | 418 ± 63 |  |  |
| Aridos 1 |  | Spain | MIS 9 | 350-300 | Biochronology |  |
| Ambrona | Upper Member | Spain | MIS 11 | < 350 | ^230^Th/^234^U |  |
|  |  |  | MIS 11 | 366 + 55/ -51 | ESR/U series |  |
|  |  |  | MIS 9 | 314 +48/ - 45 |  |  |
| Domeny |  | Spain | MIS 9 | < 317 ± 49 | ^40^Ar-^39^Ar |  |
| Puig den Roca III |  | Spain | MIS 9 | > 317 ± 49 | ^40^Ar-^39^Ar |  |
| Le Pucheuil | unit B | France | late MIS 7/ early MIS 6 | 200- 180 | Geology |  |
|  | unit A-C |  | late MIS 8/ early MIS 7 | 250 | Geology |  |
| Yiewsley Area |  | UK | late MIS 8/early MIS 7 |  | Geology |  |
| Creffield Road |  |  |  |  |  |  |
| Baker’s Hole |  |  |  |  |  |  |
| Ebbsfleet |  |  |  |  |  |  |
| Lion Pit |  |  |  |  |  |  |
| Maastricht-Belvédère | Unit IV | Netherlands | MIS 7 | 220 ± 40 | ESR |  |
|  |  |  | MIS 8 | 270 ± 22 | TL |  |
| Le Rissori |  | France | MIS 7 |  | Geology |  |
| Therdonne |  | France | MIS 7 | 178 ± 11 | TL |  |
| Biache San Vast | level IIa | France | MIS 8 | 263 +53/ -37 | ESR |  |
|  |  |  | MIS 7 | 229 ± 27 | ESR/U-series |  |
|  |  |  | MIS 7 | 230 ± 24 |  |  |
|  |  |  | MIS 6 | 175 ± 13 | TL |  |
| Bapaume Les Osiers |  | France | late MIS 7/ early MIS 6 | 195 | IRSL |  |
| Barbas | level 3 | France | MIS 6 | 146 ± 29 | TL |  |
|  |  |  |  | 147 ± 28 |  |  |
|  | level 4 |  | MIS 7 | 239 ± 44 |  |  |
| Achenheim | level 15-17a | Germany | MIS 6 |  | Geology |  |
|  | level 20a |  | late MIS 8/ early MIS 7 | 278 ± 36 | TL |  |
|  |  |  |  | 244 ± 31 |  |  |
| Gran Rois |  | France | MIS 7a |  | Geology |  |
| Cantalouette I | unit IV | France | MIS 7 | 223 ± 20 | TL |  |
| Campsas |  | France | MIS 7b |  | Geology |  |
| Torralba |  | Spain | MIS 7 | > 202 ± 18 | ^234^U/^238^U |  |
|  |  |  |  | > 243 ± 18 | ^230^Th/^234^U |  |
| Galeria Pesada | level B2 | Portugal | MIS 7 | 241 ± 22 | ESR/U series |  |
| Korolevo | level Vb | Ukraine | MIS 7 | 220 ± 35 | OSL |  |
| Le Cotte St. Brelade |  | France | MIS 7 | 238 ± 35 | TL |  |
| Salouel |  | France | MIS 6 | < 200 ± 57 | ESR |  |
| Ariendorf 1 |  | Germany | MIS 7 | > 220 | Geology |  |
| Markkeleeberg |  | Germany | MIS 6 | > 150 | Geology |  |
| Suard | level 51 | France | MIS 6 | 126 ± 15 | TL |  |
| Coudoulous I | unit 4 | France | MIS 6 | 142 ± 10 | ESR/U series |  |
|  |  |  |  | 147 ± 13 |  |  |
|  |  |  |  | 151 ± 11 |  |  |
|  |  |  |  | 154 ± 15 |  |  |
|  |  |  |  | 168 ± 16 |  |  |
|  |  |  | MIS 7 | 206 ± 20 |  |  |
| Vaufrey | level VII | France | MIS 6 | 158 ± 10 | U series |  |
|  | level IX |  | MIS 7 | 208 ± 8 |  |  |
| Baume Bonne | unit IV | France | MIS 6 | 135-125 | ESR/U series |  |
|  | unit III |  | late MIS 8 |  |  |  |
| Lazaret | Unit C | France | MIS 5 | 114 ± 10 | ESR |  |
|  |  |  | MIS 5 | 125 ± 13 |  |  |
|  |  |  | MIS 5 | 126 ± 14 |  |  |
|  |  |  | MIS 7 | 205 ± 16 |  |  |
|  |  |  | MIS 5 | 70-130 | U/Th |  |
| Lezetxiki | level VII | Spain | MIS 6 | 140 ± 6 | U/Th |  |
|  |  |  | MIS 7 | 200 + 142/ - 52 | U/Th |  |
|  |  |  | MIS 7 | 225 ± 40 | U/Th |  |
| Carihuela | level D0 | Spain | MIS 6 | 117 ± 41 | ESR |  |
|  |  |  |  | 146 ± 17 |  |  |
| Solana del Zamborino |  | Spain | MIS 6 |  | Biochronology |  |
| Krapina | level 1 | Croatia | MIS 6 | 133 ± 15 | ESR |  |
| Scario | Level 8 | Italy | MIS 6 | > 135 ± 11 | U/Th |  |
| Poggio | level 17 | Italy | MIS 5c | 111.8 | TL |  |

Dataset S1: List of the sites discussed in the paper.

**References**

1. White M, Ashton N (2003) Lower Palaeolithic Core Technology and the Origins of the Levallois Method in North-Western Europe. Current Anthropology 44: 598-609.

2. Ryssaert C (2006) Lithische technologie te Mesvin IV: selectie criteria voor geretoucheerde werktuigen en hun relatie met Levalloise in producten. Anthropologica et Praehistorica 117: 13-34.

3. van Baelen A, Meus EPM, van Peer P, de Warrimont J-P, de Bie M (2007) An early Middle Palaeolithic site at Kesselt-op de Schans (Belgian Limburg). Notae Praehistoricae 27: 19-26.

4. Schäfer J, Laurat T, Kegler J (2004) Neue archäologische Untersuchungen in Markkleeberg, Tagebau Espenhain (Lkr. Leipziger Land). Praehistoria Thuringica 10: 141-170.

5. Moncel M-H, Combier J (1992) L’outillage sur éclat dans l’industrie lithique du site Pléistocène moyen D’Orgnac 3 (Ardèche, France). L’Anthropologie 96: 5-48.

6. Shen G (1985) Datation des planchers stalagmitiques de sites acheuléens en Europe par les méthodes des déséquilibres des familles del’Uranium et contribution méthodologique.: Université de Paris VI.

7. Falguères C, Shen G, Yokoyama Y (1988) Datation de l'aven d'Orgnac III : comparaison par les méthodes de la Résonance de Spin Electronique (ESR) et du déséquilibre des familles de l'uranium. L’Anthropologie 92: 727-729.

8. Jarry M., Colonge D, A. LL, V. M (2007) Les Bosses (Lamagdelaine, Lot, France): un gisement paléolithique moyen antérieur à l'avant-dernier Interglaciaire sur la moyenne terrasse du Lot. Paris: Société Préhistorique Française.

9. Falguères C, Bahain J-J, Saleki H (1997) U-Series and ESR Dating of Teeth from Acheulian and Mousterian Levels at La Micoque (Dordogne, France). Journal of Archaeological Science 24: 537-545.

10. Colonge D, Jarry M, Delfour R, Fondeville C, Arnoux T, et al. (2010) De la transition paléolithique inférieur-moyen dans la vallée de la Garonne: l'Acheuléen supérieur de Raspide 2 (Blagnac, Haute-Garonne). Paris: Société préhistorique française. 21 p.

11. Bourguignon L, Djema H, P. B, Lahaye C, Guibert P (2008) Le gisement saalien de Petit-Bost (Neuvic-sur-l’Isle, Dordogne, tracé autoroutier A89): à l’origine du Moustérien aquitain ? In: Jaubert J, Bordes J-G, Ortega I, editors. Les sociétés du Paléolithique dans un grand Sud-Ouest de la France : nouveaux gisements, nouveaux résultats, nouvelles méthodes Actes des journées SPF Université Bordeaux-I Talence 24-25 novembre 2006. Paris: Société préhistorique française. pp. 41-55.

12. Berger GW, Pérez-González A, Carbonell E, Arsuaga JL, Bermúdez de Castro JM, et al. (2008) Luminescence chronology of cave sediments at the Atapuerca paleoanthropological site, Spain. Journal of Human Evolution 55: 300-311.

13. Falguères C, Bahain J-J, Yokoyama Y, Arsuaga JL, Bermudez de Castro JM, et al. (1999) Earliest humans in Europe: the age of TD6 Gran Dolina, Atapuerca, Spain. Journal of Human Evolution 37: 343-352.

14. Santonja M, Villa P (1990) The Lower Paleolithic of Spain and Portugal. Journal of World Prehistory 4: 45-94.

15. Ordónez S, González JA, García del Cura MA (1990) Datación Radiogénica (U^234^/U^238^ y Th^230^/U^234^) de sistemas travertínicos del Alto Tajo (Guadalajara). Geogaceta 8: 53-56.

16. Falguères C, Bahain J-J, Pérez-González A, Mercier N, Santonja M, et al. (2006) The Lower Acheulian site of Ambrona, Soria (Spain): ages derived from a combined ESR/U-series model. Journal of Archaeological Science 33: 149-157.

17. Carbonell E, Rodríguez XP (2007-2008) El Paleolítico inferior de Cataluña. Veleia 24-25: 331-343.

18. Ropars A, Billard C, Delagnes A (1996) Présentation générale de l’opération et des données archéologiques. In: Delagnes A, Ropars A, editors. Paléolithique moyen en Pays de Caux (Haute-Normandie). Paris: Éditions de la Maison des Sciences de l’Homme. pp. 28-49.

19. Scott B, Ashton N, Lewis SG, Parfitt SA, White M (2011) Technology and Lanscape Use in the Early Middle Paleolithic of the Thames Valley. In: Ashton N, Lewis SG, Stringer CB, editors. The Ancient Human Occupation of Britain. Amsterdam: Elsevier. pp. 67-89.

20. Vandenberghe J, Roebroeks W, van Kolfschoten T (1993) Maastricht-Belvédère: Stratigraphy. Palaeoenvironment and Archaeology of the Middle and Late Pleistocene Deposits. Part II. Mededelingen Rijks: Geologische Dienst

21. Adam A (2002) The pseudo-Levallois points of the Mousterian site Le Rissori at Masnuy-Saint-Jean (Hainaut, Belgium). L'Anthropologie 106: 695-730.

22. Locht J-L, Antonie P, Hérisson D, Gadebois G, Debenham N (2010) Une occupation de la phase ancienne du Paléolithique Moyen à Therdonne (Oise). Gallia Préhistorie 52: 1-32.

23. Yokoyama Y (1989) Direct gamma-ray spectrometric dating of Anteneandertalian and Neandertalian remains. In: Giacobini G, editor. Hominidae. Turin: Proceedings of the 2nd intern. Congress of Human Paleontology. pp. 387-390.

24. Bahain JJ (2007) La méthode de datation par résonance de spin électronique (ESR) au Muséum national d’histoire naturelle. Vingt ans de recherches méthodologiques et d’applications géochronologiques: Bordeaux 3. 88 p.

25. Sommé J, Tuffreau A, Aikten MJ, Auguste P, Chaline J, et al. (1988) Chronostratigraphie, climats et environnements. In: Tuffreau A, Sommé J, editors. Le gisement paléolithique moyen de Biache-Saint-Vaast (Pas-de-Calais) Stratigraphie, environnement études archéologiques Paris: Mémoires de la Société Préhistorique Française. pp. 115-119.

26. Balescu S, Tuffreau A (2004) La phase ancienne du Paléolithique moyen dans la France septentrionale (stades isotopiques 8 à 6): apports de la datation par luminescence des séquences loessiques. Archaeological Almanac 16: 5-22.

27. Boëda É, Kervazo B, Mercier N, Valladas H (1996) Barbas C’3 base (Dordogne), une industrie bifaciale contemporaine des industries du Moustérien ancien : une variabilité attendee. In: Bietti A, Grimaldi S, editors. Proceedings of the International Round Table: Reduction processes (chaînes opératoires) for the European Mousterian. Rome. pp. 465-504.

28. Richter J (2011) When Did the Middle Paleolithic Begin? In: Conard NJ, Richter J, editors. Neanderthal Lifeways, Subsistence and Technology: Springer Netherlands. pp. 7-14.

29. Buraczynski J, Butrym J (1987) Thermoluminescence stratigraphy of the loess in the southern Rhinegraben. In: Pesci M, editor. Loess and Environment: Catena Supplement. pp. 81-94.

30. Soriano S (2000) Outillage bifacial et outillage sur éclat au Paléolitique ancien et moyen: coexistence et interaction. Paris: Paris X. 460 p.

31. Brenet M, Folgado M, Lenoble A, Bertran P, Vieillevigne E, et al. (2008) Interprétation de la variabilité technologique de deux industries du Paléolithique moyen ancien du Bergeracois : Cantalouette 1 et Combe Brune 3 (Creysse, Dordogne). Contexte géoarchéologique et chronologique, analyse techno-économique. In: Jaubert J, Bordes J-G, Ortega I, editors. Les sociétés du Paléolithique dans un grand Sud-Ouest de la France : nouveaux gisements, nouveaux résultats, nouvelles méthodes Actes des journées SPF Université Bordeaux-I Talence 24-25 novembre 2006. Paris: Société préhistorique française. pp. 57-81.

32. Jaubert J, Servelle C (1996) L’Acheuléen du Bassin de la Garonne. État de la question et implications. In: Tuffreau A, editor. L’Acheuléen dans l’Ouest de l’Europe, Papers presented at the Saint-Riquier international symposium (June 6th–10th 1989). University of Sciences & Technology of Lille (1996): CERP. pp. 77-108.

33. Santonja M, Villa P (2006) The Acheulian of Western Europe. In: Goren-Imbar N, Sharon, G. , editor. Axe Age Acheulian Tool-making from Quarry to Discard Approaches to Anthropological Archaeology. London: Equinox. pp. 429-478.

34. Marks AE, Brugal J-P, Chabai VP, Monigal K, Goldberg ED, et al. (2002) Le gisement pléistocène moyen de Galeria Pesada (Estrémadure, Portugal) : premiers résultats. PALEO 14: 77-100.

35. Haesaerts P, Koulakovskaya LV (2006) La séquence paléosedimentaire de Korolevo (Ukraine transcarpathique): contexte chronostratigraphique et chronologique. In: Koulakovskaya LV, editor. The European Middle Paleolithic. Kiev: Shlyakh. pp. 21-38.

36. Callow P, Conford JM (1986) La Cotte de St. Brelade 1961-1978: Excavation by C. B. M. McBurney. Noewich: Kluwer Academic Publishers.

37. Ameloot-van der Heijden N, Dupuis C, Limondin N, Munaut AV, Puissegur JJ (1996) The Middle Palaeolithic open air site of Salouel. L'Anthropologie 100: 555-573.

38. Blackwell B, Schwarcz HP, Debénath A (1983) Absolute dating of hominids and palaeolithic artifacts of the cave of La Chaise-de-Vouthon (Charente), France. Journal of Archaeological Science 10: 493-513.

39. Jaubert J, Kervaso B, Bahain J-J, Brugal J-P, Chalard P, et al. (2005) Coudoulous I (Tour-de-Faure, Lot), site du Pléistocène moyen en Quercy : bilan pluridisciplinaire. In: Molines N, Moncel M-H, Monnier J-L, editors. Les premiers peuplements en Europe : Données récentes sur les modalités de peuplement et sur le cadre chronostratigraphique, géologique et paléogéographique des industries du Paléolithique ancien et moyen en Europe (Rennes, 22-25 septembre 2003). Oxford: British Archaeological Reports. pp. 227-252.

40. Blackwell B, Schwarcz HP (1988) Datations des spéléothems de la grotte Vaufrey par la famille de l’uranium. In: J.P. R, editor. La Grotte Vaufrey: Paléoenvironnement, chronologie, activités humaines. Paris: Mémoires de la Société Préhistorique Française pp. 365-380.

41. Falguères C, Laurent M, Ajaja O, Bahain J-J, Yokoyama Y, et al. (1993) Datation par les méthodes U-Th et ESR de la grotte de la Baume Bonne (Alpes-de Haute Provence, France). Actes du 12ème Congrès UISPP Bratislava, 1-5 septembre 1991. pp. 98-107.

42. Michel V, Shen G, Valensi P, de Lumley H (2009) ESR dating of dental enamel from Middle Palaeolithic levels at Lazaret Cave, France. Quaternary Geochronology 4: 233-240.

43. Falguères C, de Lumley H, Bischoff JL (1992) U-Series dates for stalagmitic flowstone E (Riss/Würm interglaciation) at Grotte du Lazaret, Nice, France. Quaternary Research 38: 227-233.

44. Baldeon A (1993) El yacimientos de Lezetxiki (Gipuzkoa, Pais Vasco). Los nivel musterienses. Munibe (Antropologia-Arkeologia) 45: 3-97.

45. Falguères C, Yokoyama Y, Arrizabalaga A (2005) La geocronologia del yacimiento de Lezetxiki (Arrasate, Pais Vasco). Critica de las dataciones existentes y algunas nuevas aportaciones. Munibe (Antropologia-Arkeologia) 57: 93-106.

46. Vega Toscano LG, Villar A, Rojas T, Cosano P, Escarpa O (1996) Las industrias de la interfase Pleistoceno medio-superior en la Cueva de la Carihuela (Piñar, Granada). In: Ramírez PB, Behemann BR, editors. II Congreso de Arqueologia Peninsular. pp. 105-118.

47. Caro Gómez JA, Díaz Del Olmo F, Artigas RC, Recio Espejo JM, Barrera CB (2011) Geoarchaeological alluvial terrace system in Tarazona: Chronostratigraphical transition of Mode 2 to Mode 3 during the middle-upper pleistocene in the Guadalquivir River valley (Seville, Spain). Quaternary International 243: 143-160.

48. Rink WJ, Schwarcz HP, Smith FH, Radovcic J (1995) ESR ages for Krapina hominids. Nature 378: 24.

49. Ronchitelli A, Boscato P, Surdi G, Masini F, Petruso D, et al. (2011) The Grotta Grande of Scario (Salerno, Italy): Archaeology and environment during the last interglacial (MIS 5) of the Mediterranean region. Quaternary International 231: 95-109.

50. Boscato P, Boschian G, Caramia F, Gambassini P (2009) Il Riparo del Poggio a Marina di Camerota (Salerno): culture ambiente. Rivista di Scienze Preistoriche LIX: 5-40.
