# Supplementary material for: San Bernardino Cave (Italy) and the Appearance of Levallois Technology in Europe: Results of a Radiometric and Technological Reassessment
Source: PLoS One. 2013 Oct 16;8(10):e76182. doi: 10.1371/journal.pone.0076182 (PMC3797834; doi:10.1371/journal.pone.0076182)
Supplement: Text S1 — Archaeological Context. (DOC) [file pone.0076182.s016.doc]

**Archaeological Context**

Grotta Maggiore di San Bernardino is located in the northern-east of the Italian Peninsula, 25 km south of Vicenza. The cave is situated on the eastern slope of the Berici karst plateau, at 135 m above sea-level, facing the alluvial plain of Bacchiglione river and the south areas of Euganei Hills (Figure S1). In the western side of the same slope is situated a second shorter cavity named San Bernardino Minor Cave. The karst system decreases gently to the south-west whereas in the eastern boundary connects abruptly with the marshy plain, rich of peat deposits. On this side of the Berici Hills opens many caves, beside San Bernardino Cave, that yielded abundant Upper Pleistocene archaeological evidences as Paina Cave and Broin Cave .

The natural morphology of San Bernardino Cave has been modified during the Middle Ages widening the inner part and building a wall at the entrance and during the Modern Ages when the internal fill was partially destroyed. The first archaeological excavations were carried out by Prof. Leonardi in the ’60 in the medieval wall facing area, uncovering a Pleistocene sequence with faunal remains and lithic assemblages . A second cycle of researches, between 1986 and 1995, allowed a more detailed reexamination of the stratigraphic series of the different facies between the inner and outer zones of the cave.

**Stratigraphy**

The entrance of the cave preserves a sequence currently visible on the longitudinal section exposed during the new excavations and constituted by 8 lithological units (Figure S2):

**Unit I**: visible in the unique testimony on the Northern wall of the cave it is composed of thermoclastic breccia and loess matrix with big angular stones, few lithic and faunal remains; the lower limit is abrupt.

**Unit II**: situated at the entrance of the cave and occupied the area below the medieval wall that lies on it. The deposition includes medium size sub-rounded stones associated with dark sandy-lime soil with organic matter. The unit is rich in faunal remains, lithic assemblages and hearths.

**Unit III**: it covers the same area occupied by unit II and comprehends calcareous pebbles and lithic artifacts, rounded by stripping of water. This unit lies on an undulate surface of erosion.

**Unit IV**: situated in the front of the cave entrance, the deposit is composed of light dark loess, devoid of stones and highly calcareous. Faunal and lithic remains are scarce. The lower limit is gradual.

**Unit V**: include the whole excavation surface and it is composed of cryoclastic breccia with big angular stones on loessic matrix with few lithic and faunal remains. The stones in correspondence of the entrance area show a horizontal disposition whereas in the more exterior area assumed chaotic dispositions for the cryoturbation and solifluction processes; the inferior limit is evident.

**Unit VI**: include the whole excavation surface. The anthropogenic unit with hearths is composed of sub-rounded stones of medium size associated to dark silty-sand matrix with organic material; in the interior side the soil is siltier with frequent rusted stones; the exterior side is characterized by the formation of a paleosoil. The lower limit is evident.

**Unit VII**: in the atrial zone of the cave lies on natural rock step. It is constituted by cryoclastic breccia with big angular stones and few faunal and lithic remains. In the inner side are preserved hearths with flints and bones. The lower limit is abrupt.

**Unit VIII**: the extension is lower than unit VII. It is composed of alternated centimetric anthropogenic strata and colluvium of gravel and calcareous granules.

**Results**

**Chronology**

The main difficulty of ESR dating of fossil teeth and bones is the open system behavior of the tissues for the U-series isotopes. Uranium uptake can be modeled by combining U-series and ESR data (ESR-US), using the following function: U(t) = U_m_ (t/T)^p+1^, where U(t) is the uranium concentration at the time t, U_m_ is the measured, present day U-concentration, T is the age of the sample and p is the uptake parameter . Frequently used parametric U-uptake models are special cases of the ESR-US model: a p-value of -1 corresponds to the Early Uptake (EU) model (closed system assumption), p=0 to the Linear Uptake (LU) model and p>>1 approximates the Recent Uptake (RU) model (see ), , and for basic concepts of the respective models). The potential and limitation of the combined ESR-US approach, particularly for fossil bones dating were discussed by .

**Samples**

Five bones from Unit VII and one herbivorous tooth and two bones from units VIII were analyzed with ESR-US technique. The samples generally did not exhibit much alteration and were sufficiently in good state to be analyzed. Table S2 shows the layers and squares from where they were found.

**Experimental**

Sample preparation, irradiation and ESR measurements followed the analytical procedures usually applied in the Paris lab. (see details in ; ). The ESR intensities were extracted from peak-to-peak amplitudes of the ESR signal of enamel (T1-B2; see ). The experimental data points were fitted with a single saturating exponential (SSE) function weighting the data by the inverse of the squared intensity .

The U-series isotopes in dental tissues and bones were analyzed with alpha-ray spectrometry (Table S1). Enamel was analyzed by alpha-ray spectrometry using the standard procedures of . U-concentrations of cement and dentine and bones were assessed by gamma spectrometry . All analyses were carried out at the Department of Prehistory of the Museum National d’Histoire Naturelle (France).

Combined ESR-US ages were calculated with the DATA program using the following parameters: an alpha efficiency of 0.13+0.02 , Monte-Carlo beta attenuation factors from Brennan et al. (1997), dose-rate conversion factors from , a water content 5 ± 3 wt% in dentine and cement and 10 ± 5 wt% in sediments. Ra and Rn loss in each tissue was determined by gamma-ray spectrometry . The gamma dose rate was derived by averaging *in situ* dose rates measured by TL dosimeters (Ca SO4:Dy), plus laboratory values obtained from the radioisotope analysis (U, Th and K) of the sediments surrounding the teeth . The cosmic dose rate was calculated according to . P-values, very closed from -1, are obtained for SB27, SB28, SB33 and SB35. For the other samples for which it was not possible to calculate an ESR-US age, we used a model called Accelerating Uptake model (AU) able to reconstruct a process combining uranium incorporation followed by leaching . The AU model yields minimum ages (Table S2).

A mean age of 181 ka suggests that the Units VIII and VII were deposited during the end of MIS7 and the beginning of MIS6.

**Technological analyses**

**Raw Material**

The raw material used in the lithic assemblages of Unit VIII and Unit VII of San Bernardino Cave is flint, gathered principally in primary deposition outcrops. Fluvial pebbles have not been recovered. At close distance from the site (1-2 km) outcrop the Scaglia Rossa lithological type, a reddish-brown microcrystalline flint. The abundance of calcareous inclusions and natural fractures make these local nodules mediocre for knapping. The exogenous raw materials instead are composed of microcrystalline grey flints, named Maiolica and Scaglia Variegata. The nodules are homogenous and compact with higher flaking qualities. The outcrops are located in the Euganean Hills (10-20 km) and at longer distance in the Lessini Mountains (80 km). Another exogenous raw material originates in the Tertiary Formation of the Lessini Mountains and in some localities of the Euganean Hills.

**Unit VIII**

The lithic assemblage of Unit VIII is composed of 9596 lithic items and comprises 470 flakes (4.9%), 43 retouched tools (0.4%) and 22 cores (0.2%) (Table S3). The raw material most utilized is Scaglia Rossa (96%) while the lithological types Maiolica (3%), Scaglia Variegata (0.01%) and Tertiary Formation flints (1%) are used in lesser percentages. Small nodules were configured at the site since the entire operative chain is present (Table S4). The Levallois recurrent centripetal is the dominant flaking modality even if very few recurrent unidirectional flakes (0.4%) and one centripetal core were recovered (Table S4 and S5, Figure S3). The absence of raw or at least partially modeled cobbles does not provide any evidence for reconstructing the onset of the core reduction sequence. Conversely, cortical flakes show that natural convexities were exploited as well at the place of consuming flint for carefully shaping the future core face. Core exploitation involves removing of predetermined/ing Levallois flakes following *linear or crossed* sequences in alternance with core management. The peripheral core convexities are prepared by detaching naturally backed flakes, pseudo-Levallois points and core-edge flakes, step by step after each single or a short series of Levallois flakes (Table S4). The same holds for the core platforms, which are mostly faceted over 60% of the perimeter. Therefore, the combined removals of ordinary and Levallois core-edge flakes with the predetermined centripetal flakes auto-maintains the core face, conformably to a continuous process. These flakes bear a natural back with opposed thin edge. In some cases, the back shows remnants of faceted core platforms. Although, due to these options, it was possible to produce several blanks per core, their morpho-metrical and morpho-technical standard appears low in terms of potentially functional edges. Sometimes, predetermination invested the proximal portion of the flaking core-face in order to remove prominent residual ridges or regularize the outline and the thickness of the Levallois flake. This intervention involved the detachment of one or more marginal tiny flakes around the impact point of the predetermined flake and before the final preparation of the butt. Small hard hammers were used for percussion.

In the assemblage were found also predetermined flakes categorized as indeterminate (2.8%) because the dorsal scars patterns do not allow a precise attribution to the Levallois modality recurrent centripetal or unidirectional..

Secondary *chaînes opératoires* include centripetal (21.2%) and polyhedral methods (3%), and core-on-flakes (39.4%) (Table S5, Figure S4). In the assemblage within centripetal (0.9%) and laminar (1.9%) blanks, are present also unidirectional (1.3%) and orthogonal flakes (0.4%) (Table S4). The small number of secondary *chaînes opératoires* products suggests a technological shift to more expeditious methods when natural fractures or calcareous inclusion impeded the continuation of the Levallois recurrent centripetal production. Cores are exhausted and abandoned at the reach of similar dimension.

Cores-on-flakes are of small dimension and with limited production potentiality. The technological analysis recognized two main different patterns of reduction. In the first group were identified 9 core-on-flakes characterized by the exploitation of the flake ventral surface (Figure S4 nº4). The blanks utilized are mostly semi-cortical and the direction of the scars indicates a higher frequency of centripetal pattern than convergent unidirectional. The second group is composed of 4 cores-on-flakes distinguished by the prismatic morphology of the blanks and characterized by unidirectional or orthogonal exploitation in order to achieve elongated flakes (Figure S4 nº5). These laminar products (1.9%) are few in the assemblage (Table S4).

Retouched tools are composed principally of scrapers (58.1%), convergent scrapers (14%) and denticulates (14%) whereas notched tools (7%) and multiple tools (7%) are present in lesser percentages (Table S6). Although some stone tools were extensively retouched, impeding their technological reading, in the remaining part of the assemblage is absent a preference for a particular morphology or dimension of the blanks (Figure S5).

**Unit VII**

The lithic material of Unit VII is composed of 4137 lithic artifacts and comprises 230 flakes (5.6%), 19 cores (0.5%) and 29 retouched tools (0.7%) (Table S3). The local raw material Scaglia Rossa (96.3%) is the most utilized while the exogenous flint Maiolica (3.4%), Scaglia Variegata (0.1%) and Tertiary formation flint (0.2%) is used in lesser percentage. The technological analysis recognized that the knapping sequences were carried out at the site since all the technological categories are present (Table S4). The Levallois method is identified in 1 core in the modality preferential while the recurrent scheme is documented only by unidirectional (4.8%) and centripetal products (2.2%) (Table S4, Figure S6). Cortical flakes show that natural convexities were exploited for the same purpose inferred in unit VIII. The distal convexity of the face core was drawn by natural inclined surfaces. Sometimes, at the early stage Levallois cores were exploited by means of orthogonal pattern in the same manner as described by . Many different blanks served to maintain the core lateral convexities and an equilibrated morphology of the core face. Among these products, Levallois core-edge flakes are frequent (Table S4). Ordinary predetermining Levallois flakes (16.5%) are abundant as well (Table S4). These items shape the lateral convexities by means of marginal detachments. The distal convexity was maintained as well by means of trimming-core flakes, trimming partially plunged flakes or marginal flakes detached from an opposed striking platform. The operative schemes show that unipolar Levallois detachments followed a *leftward or rightward* sequence of two or sometimes more predetermined blanks, each of which was removed alongside the former one in order to exploit both the ridges and the lateral convexity drawn by these wide scars. In those cases where the impact points overlie one another, flake removal required a preliminary preparation of the lateral convexity. As seen in unit VIII, predetermination invested also the proximal portion of the flaking core-face in order to remove prominent residual ridges or ameliorate the outline and the thickness of the Levallois flake. The preparation of the striking platform increased with a higher number of faceted and winged morphologies in the Levallois recurrent unidirectional products while in recurrent centripetal blanks faceted and dihedral forms are maintained. As regards recurrent centripetal flakes, technological features show that the convexities were managed both by Levallois and ordinary core-edge flakes and by predetermining marginal flakes.

Secondary *chaînes opératoires* include unidirectional (26.1%), centripetal (26.1%) and laminar (4.3%) flaking strategies (Table S5, Figure S7). The comparison between the number of centripetal (3%) and unidirectional flakes (3.5%) and the amount of cores suggests that these more expeditious methods were applied in later stages of reduction or when the inclusion of the raw material impeded the maintenance of the hierarchical division of the core volume. Within the unidirectional reduction was recognized the production of big flakes using salami slices method . This strategy permitted the regularization of the core surfaces from natural fractures or calcareous inclusion. Sometimes these large blanks were suitable to produce scrapers. The laminar reduction sequence is present with a prismatic core, characterized by orthogonal removals (Figure S7 nº4), and few laminar products (3.5%) (Table S4). Cores-on-flakes are few (21.7%) (Table S5) and typified by the exploitation of the flake ventral surface with centripetal or unidirectional scar patterns.

The assemblage of retouched tools comprehends mainly scrapers (51.7%) whereas notched tools (13.8%) and denticulates (24.1%) are present in lesser percentages (Table S6, Figure S8). The blanks modified by retouch are principally semi-cortical flakes and only three Levallois flakes were transformed in scrapers. Two scrapers and one denticulate were thinned on the ventral surface on the opposite side of the working edge.

**References**

1. Leonardi P, Broglio A (1961-1962) Il Bernardiniano, nuova industria litica musteriana. Atti Istituto Veneto SSLLAA pp. 261-283.

2. Leonardi P (1958-1959) Risultati della prima campagna di scavo della stazione musteriana di S.Bernardino nei Colli Berici (VI). Atti Istituto Veneto SSLLAA pp. 387-402.

3. Grün R, Schwarcz HP, Chadam J (1988) ESR dating of tooth enamel: Coupled correction for U-uptake and U-series disequilibrium. International Journal of Radiation Applications and Instrumentation Part D Nuclear Tracks and Radiation Measurements 14: 237-241.

4. Bischoff JL, Rosenbauer RJ (1981) Uranium series dating of human skeletal remains from the Del Mar and Sunnyvale sites, California. Science 213: 1003-1005.

5. Ikeya M (1983) ESR Studies of Geothermal Boring Cores at Hachobara Power Station. Japanese Journal of Applied Physics 22: 763-765.

6. Blackwell BN, Porat N, Schwarcz HP, Debenath A (1992) ESR dating of tooth enamel : comparison with 230Th / 234U speleothem dates at La Chaise-de-Vouthon (Charente), France. Quaternary Science Reviews 11: 231-244.

7. Grün R, Aubert M, Hellstrom J, Duval M (2010) The challenge of direct dating old human fossils. Quaternary International 223–224: 87-93.

8. Falguères C, Bahain J-J, Yokoyama Y, Arsuaga JL, Bermudez de Castro JM, et al. (1999) Earliest humans in Europe: the age of TD6 Gran Dolina, Atapuerca, Spain. Journal of Human Evolution 37: 343-352.

9. Bahain J-J, Laurent M, Falguères C, Voinchet P, Farkh S, et al. (2002) Datation par résonnance paramagnétique électronique (RPE) des formations fluviatiles pléistocènes et des gisements archéologiques ou paléontologiques associés. Quaternaire: 91-103.

10. Grün R (2000) Methods of dose determination using ESR spectra of tooth enamel. Radiation Measurements 32: 767-772.

11. Yokoyama Y, Falgueres C, Quaegebeur JP (1985) ESR dating of quartz from quaternary sediments: First attempt. Nuclear Tracks and Radiation Measurements (1982) 10: 921-928.

12. Bischoff JL, Rosenbauer RJ, Tavoso A, de Lumley H (1988) A test of uranium-series dating of fossil tooth enamel: results from Tournal Cave, France. Applied Geochemistry 3: 145-151.

13. Yokoyama Y, Nguyen HV (1980) Direct and non destructive dating of marine sediments, manganese nodules and corals by high resolution gamma-ray spectrometry. In: Goldberg ED, Horibe Y, Saruhashi K, editors. Isotope marine chemistry. Tokyo: Uchida Rokakuho. pp. 259-289.

14. Grün R (2009) The DATA program for the calculation of ESR age estimates on tooth enamel. Quaternary Geochronology 4.

15. Grün R, Katzenberger-Apel O (1994) An alpha irradiator for ESR dating. Ancient TL 12: 35-38.

16. Adamiec G, Aikten MJ (1988) Dose-rate conversion factors: update. Ancient TL 16: 37-50.

17. Bahain JJ, Yokoyama Y, Falguères C, Sarcia MN (1992) ESR dating of tooth enamel: a comparison with K-Ar dating. Quaternary Science Reviews 11: 245-250.

18. Prescott JR, Hutton JT (1988) Cosmic ray and gamma ray dosimetry for TL and ESR. International Journal of Radiation Applications and Instrumentation Part D Nuclear Tracks and Radiation Measurements 14: 223-227.

19. Shao Q, Bahain J-J, Falguères C, Dolo J-M, Garcia T (2012) A new U-uptake model for combined ESR/U-series dating of tooth enamel. Quaternary Geochronology 10: 406-411.

20. Delagnes A (1992) L'Organisation de la production au Paléolithique moyen: approache technologique à partir de l'étude des industries de la Chaise-de-Vouthon (Charente) Paris: Paris X, Nanterre. 386 p.

21. Turq A (1992) Raw material and technological studies of the Quina Mousterian in the Périgord. In: Dibble H, Mellars PA, editors. The Middle Paleolithic: adaptation, behavior, and variability. Philadelphia: University of Pennysylvania, University Museum Monographs. pp. 75-85.

**Figure Legends**

Figure S1: Geographical map of the North-East of Italy with position of San Bernardino Cave (1) in the Berici Hills.

Figure S2: The stratigraphy of the San Bernardino Cave from units VIII to II. Key: 1. disturbed deposit with medieval finds; 2. bioturbation; 3. main palaeo-living floors; 4. loess; 5. thermoclastic breccia; 6. limestone gravel; 7. paleosoil.

Figure S3: Levallois Recurrent Centripetal flakes (1-7), refitting centripetal flakes (8) of Unit VIII

Figure S4: Centripetal cores (1-3) and core-on-flakes (4-5) of Unit VIII. The scar orientation is indicated by the arrow, a black dot indicates the presence of a negative bulb, and the grey color indicates the presence of cortex.

Figure S5: Retouched tools of Unit VIII.

Figure S6: Levallois Recurrent Unidirectional flakes (1-2), Levallois Recurrent Centripetal flakes (3-5), refitting core-edge flake (6) of Unit VII.

Figure S7: Centripetal (1-2), unidirectional (3) and laminar (4) cores of Unit VII.

Figure S8: Retouched tools of Unit VII.
